# Antibiotic-induced dysbiosis of gut microbiota impairs corneal development in postnatal mice by affecting CCR2 negative macrophage distribution

**DOI:** 10.1038/s41385-019-0193-x

**Published:** 2019-08-21

**Authors:** Mingjuan Wu, Jun Liu, Fanying Li, Shuoya Huang, Jingxin He, Yunxia Xue, Ting Fu, Shanshan Feng, Zhijie Li

**Affiliations:** 1 International Ocular Surface Research Center, Institute of Ophthalmology, and Key Laboratory for Regenerative Medicine, Jinan University, Guangzhou, China; 2 Department of Microbiology and Immunology, School of Medicine, Jinan University, Guangzhou, China; 3 Department of Ophthalmology, The First Affiliated Hospital of Jinan University, Guangzhou, China; 4 Department of Ophthalmology, Henan Provincial People’s Hospital, Zhengzhou, China

## Abstract

Antibiotics are extremely useful, but they can cause adverse impacts on host bodies. We found that antibiotic treatment altered the composition of the gut microbiota and the gene expression profile in the corneal tissues of postnatal mice and decreased the corneal size and thickness, the angiogenesis of limbal blood vessels, and the neurogenesis of corneal nerve fibers. The reconstitution of the gut microbiota with fecal transplants in antibiotic-treated mice largely reversed these impairments in corneal development. Furthermore, C–C chemokine receptor type 2 negative (CCR2^−^) macrophages were confirmed to participate in corneal development, and their distribution in the cornea was regulated by the gut microbiota. We propose that the CCR2^−^ macrophage population is a crucial mediator through which gut microbiota affect corneal development in postnatal mice. In addition, probiotics were shown to have the potential effect of restoring corneal development in antibiotic-treated mice. Abx-induced gut dysbiosis has significant, long-term effects on the development of the cornea, and reversal of these suppressive effects takes a long time.

## Introduction

The development of vision in newborns is incomplete and easily affected by external factors, such as a lack of nutrients, the overuse of drugs, and stimulation by high-intensity light. These adverse factors may cause the abnormal development of the eyeballs and the alteration of neurogenesis, thus permanently impairing vision.^1^ The cornea is located at the anterior segment of the eye and is responsible for nearly two-thirds of the refractive power of the optic axis. Recent studies on the development of vision have mainly focused on the retina,^2–5^ while studies on the factors that affect the development of the neonatal cornea are rare.

Because mouse eyelids are still closed for a few days post birth, mice are considered one of the optimal animal models for studying the development of neonatal corneas. The mouse cornea is still immature after birth, the epithelium has only one or two cell layers, and the stromal layer is in the translucent state. With increasing postnatal age (a matter of days), the thickness of the corneal epithelium and stromal layer gradually increase and reach the level exhibited in adult mice at postnatal day 30 (P30),^6^ while the corneal endothelium always has one cell layer.^7^ The corneal area gradually increases after birth and reaches a steady state at P60.^8^ Angiogenesis and neurogenesis are important processes in corneal development and start at embryonic day 12.5─13.5 (E12.5─13.5) in mice. The area of the limbal blood vessels gradually increases after birth and reaches the level of an adult mouse’s cornea at P21.^9,10^ Nerve fibers first grow into the anterior segment and the corneal stroma from four directions at E12.5─13.5 and then innervate the epithelium at E16.5. These epithelial nerve fibers finally gather at the corneal center to form the typical vortex at P21.^11^

Given the susceptibility of newborns to pathogens, they are usually treated with antibiotics (Abxs) if they have an infection. More than half of neonates are treated with Abxs within the first month after birth.^12^ Abxs, though useful for curing infectious diseases, bring certain adverse changes to host bodies. Some of these changes are attributed to the impacts of Abxs on microorganisms in the gastrointestinal tract, since these gut microbiota are strongly associated with health.^13,14^ Recent studies have revealed that the gut microbiota are also closely associated with the growth of infants. Schwarzer et al. confirmed that the gut microbiota affected the growth of newborns by controlling the secretion and activity of growth hormones and insulin-like growth factor (IGF).^15^ Blanton et al. transplanted the feces from healthy or malnourished children to 5-week-old, germ-free mice and found that the weight of mice that received the feces of healthy children was higher than that of mice that received the feces of malnourished children.^16^ The long-term use of Abxs, especially oral Abxs, can destroy the balance of gut microbiota and negatively impact the development of newborns. Whether the dysbiosis of gut microbiota induced by Abxs affects corneal development in postnatal mice is unclear.

Gut microbiota have been shown to help regulate the immune system. Abx-induced dysbiosis of gut microbiota in mice decreases the number of Ly6C^hi^ monocytes in the brain,^17^ and a deficiency of gut microbiota in mice affects the distribution and maturation of microglia and impairs the innate immune responses in the brain.^18^ Moreover, the resident segmented filamentous bacteria in the intestinal tract are associated with the activation of T helper 17 (Th17) cells; a deficiency in these bacteria leads to a decrease in the number and function of Th17 cells.^19^ Fusobacteria in the intestinal tract have been shown to participate in the differentiation of regulatory T cells (Tregs) from CD4^+^ T cells and to induce the expression of IL-10 in Tregs to maintain the homeostasis of immunity.^20–22^ The distribution and function of B cells are also regulated by the gut microbiota. Gut microbiota-defective mice have fewer B cells and an impaired ability to produce IgA.^23^

Macrophages, the innate immune cells, are widely distributed in peripheral tissues and are involved in the development of numerous tissues and organs. For instance, microglia can mediate innervation by regulating the survival and the programmed cell death of neurons^24^ and can secrete IGF-1 to promote the formation of the cortical V region;^25–27^ a deficiency in these cells results in a decrease in the complexity and innervation of the central nervous system (CNS).^28^ In the remodeling of bone, such as in tooth eruption and osteopetrosis, bone formation is usually impaired in macrophage-defective mice.^29,30^ Furthermore, in angiogenesis, macrophages secrete hypoxia-inducible factor (HIF-1α), which is involved in cardiovascular remodeling.^31^ In neurogenesis, macrophages secrete neurotrophin and guide the migration of Schwann cells to participate in the formation of nerve systems.^32^ It is still unclear whether macrophages participate in corneal development, and whether these cells are affected by gut microbiota.

In this study on Abx treatment, we investigated the effects of gut microbiota on corneal development in neonatal mice by observing corneal morphogenesis and the formation of limbal blood vessels and corneal nerves. We also explored the role of macrophages in corneal development and the regulation of corneal macrophages by gut microbiota.

## Results

### Abx treatment affects corneal morphogenesis

**Fig. 1:**
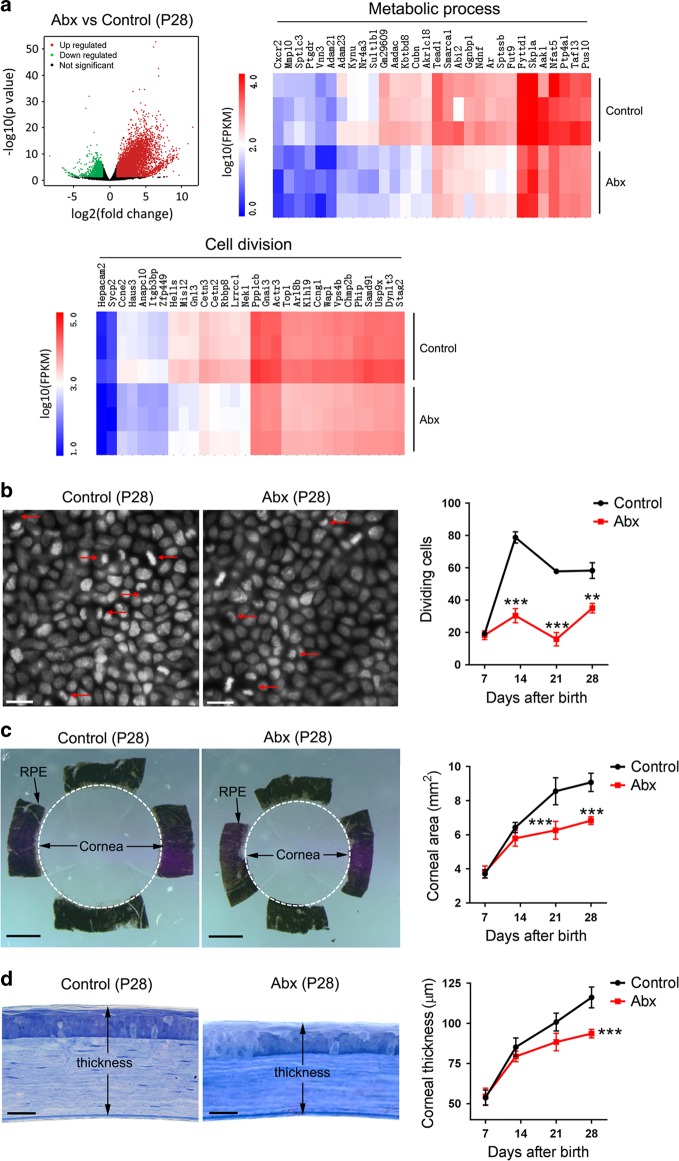
Alteration of corneal morphogenesis in postnatal mice after oral antibiotic (Abx) treatment. **a** Gene transcripts of corneal tissues in Abx-treated and control mice at postnatal day 28 (P28) were detected with RNA-sequencing (RNA-Seq) (*n* *=* 3 independent experiments, six mice per experiment in each group). The upper-left image is a volcano plot of altered genes in corneal tissues after Abx treatment; the upper-right image is a heatmap of genes involved in metabolic processes (the top 30 differentially expressed genes) (Supplementary Table 1); and the lower image is a heatmap of genes involved in cell division (the top 30 differentially expressed genes) (Supplementary Table 2). **b** Comparison of the proliferation capacity of basal epithelial cells between Abx-treated mice and control mice at P7, P14, P21, and P28. The two left images show DAPI staining of corneas from the two groups of mice (scale bars, 25 μm), and the right graph shows the dynamic changes of dividing basal epithelial cells in the two groups of mice after birth. Dividing cells were counted in zones in four quadrants of each cornea under a 60 × oil immersion lens (Supplementary Fig. 3), and the total cell number of these counts was plotted against the time after birth (*n* *=* 6 mice at each time point in each group). **c** Comparison of the corneal area between Abx-treated mice and control mice at P7, P14, P21, and P28. The two left images show the spatial range of the cornea as distinguished by the black retinal pigment epithelium (RPE) in Abx-treated and control mice (scale bars, 500 μm). The right graph shows the dynamic changes in the corneal area in the two groups of mice after birth (*n* *=* 6 mice at each time point in each group). **d** Comparison of the corneal thickness between Abx-treated mice and control mice at P7, P14, P21, and P28. The two left images show plastic embedding and toluidine blue staining of eye sections in Abx-treated and control mice (scale bars, 20 μm), and the right graph shows the dynamic changes in the corneal thickness in the two groups of mice after birth (*n* *=* 6 mice at each time point in each group). The data are presented as mean ± SD. **P < 0.01, and ***P < 0.001

After continuous broad-spectrum Abxs (ampicillin, vancomycin, neomycin sulfate, and metronidazole) treatment for 2 weeks, the gut microbiota of mice at P14 were already in dysbiosis (Supplementary Fig. 2). An RNA-sequencing (RNA-Seq) analysis showed that gene transcription in the corneal tissues of Abx-treated mice at P28 was markedly different from that in the control mice treated with sterile water; the expression of 2876 genes was downregulated, and that of 5897 genes was upregulated. Among these altered genes, 1007 genes involved in the metabolic process and 81 genes involved in cell division were downregulated (Fig. 1a). As is well known, metabolic activity is strongly associated with the developmental process.

Further investigation showed that the number of dividing epithelial basal cells in Abx-treated mice was lower than that of control mice at P14, P21, and P28 days (Fig. 1b). This means that Abx treatment decreases the proliferation capacity of corneal cells. Using the black retinal pigment epithelium (RPE), we identified the spatial range of the cornea and found that the corneal area in Abx-treated mice was smaller than that of control mice at P21 and P28 (Fig. 1c). Moreover, the thickness of cornea detected with plastic-embedding techniques and toluidine blue staining of the eye section was also decreased after Abx treatment at P28 (Fig. 1d).

### Abx treatment impairs angiogenesis in limbal blood vessels

**Fig. 2:**
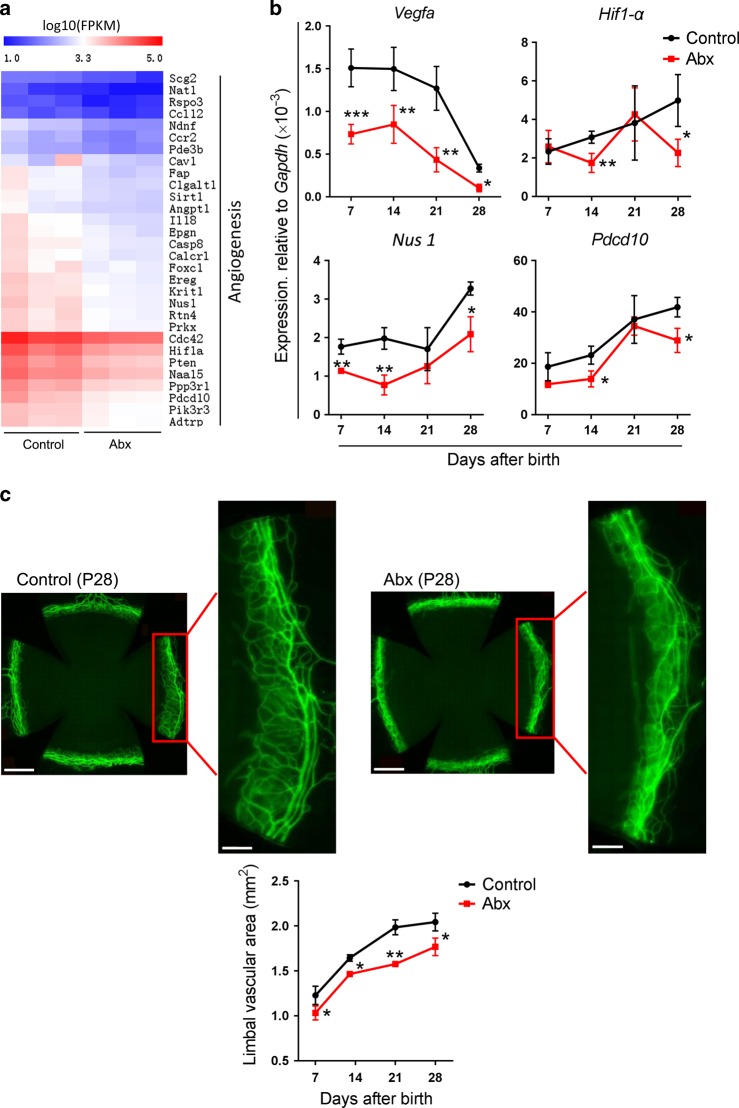
Changes in angiogenesis in the limbal blood vessels from antibiotic-treated (Abx-treated) and control mice. **a** Heatmap of genes involved in angiogenesis (the top 30 differentially expressed genes) (Supplementary Table 3) in corneal tissues of Abx-treated and control mice at postnatal day 28 (P28) (*n* *=* 3 independent experiments, six mice per experiment in each group). **b** The difference in the expression of the *Vegfa*, *Hif1a*, *Pdcd10*, and *Nus1* genes that participate in angiogenesis in corneal tissues between Abx-treated mice and control mice at P7, P14, P21, and P28 with qPCR analysis (*n* *=* 3 independent experiments, six mice per experiment in each group). **c** Comparison of the area of the limbal blood vessels in Abx-treated and control mice. The four upper images show the staining of the limbal blood vessels with anti-CD31 antibody in the two groups of mice (scale bars: the images of whole cornea, 500 μm, the partial enlarged views of limbal blood vessels, 200 μm). The lower graph shows the dynamic changes in the area of the limbal blood vessels in the two groups of mice at P7, P14, P21, and P28 (*n* *=* 6 mice at each time point in each group). The data are presented as mean ± SD. *P < 0.05, **P < 0.01, and ***P < 0.001

Angiogenesis is crucial for corneal morphogenesis, as the transport of nutriments and metabolites in corneal development requires the complete blood vessel network. RNA-Seq analysis showed that the corneal genes involved in angiogenesis of Abx-treated mice at P28 decreased compared with those of control mice (Fig. 2a). Furthermore, the expression of angiogenesis genes, the *vascular endothelial growth factor (Vegf)-a*, *hypoxia-inducible factor 1-alpha* (*Hif1-α*), *nuclear undecaprenyl pyrophosphate synthase 1* (*Nus 1*), and *programmed cell death 10* (*Pdcd10*) in the corneal tissues of Abx-treated and control mice during the postnatal development period was validated by quantitative polymerase chain reaction (qPCR) analysis, which showed that, after Abx treatment, the expression of *Vegfa* decreased at P7, P14, P21, and P28; the expression of *Hif1α* decreased at P14 and P28; the expression of *Nus1* decreased at P7, P14, and P28; and the expression of *Pdcd10* decreased at P14 and P28 (Fig. 2b). From the above results, we inferred that the formation of the limbal blood vessels may have been impaired. Therefore, we used anti-CD31 antibody immunostaining to measure changes in the blood vessel area. The results revealed that the area of the limbal blood vessels in Abx-treated mice decreased at P7, P14, P21, and P28 when compared with that of control mice (Fig. 2c).

### Abx treatment impairs corneal neurogenesis

**Fig. 3:**
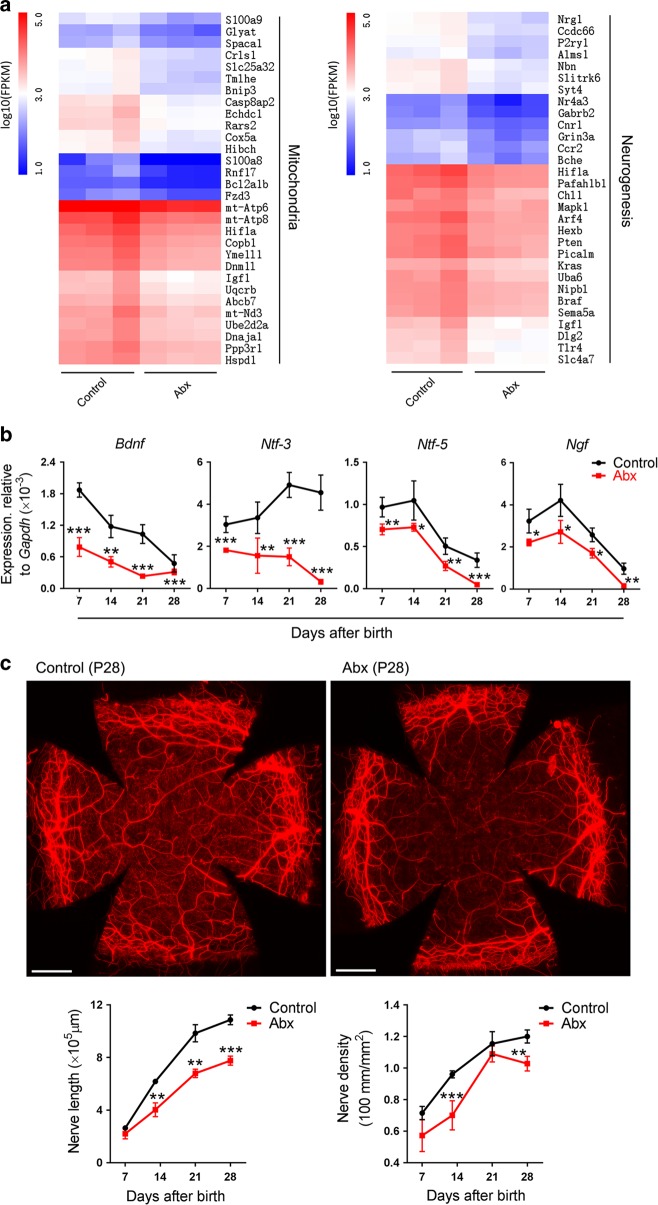
Changes in neurogenesis in corneal nerve fibers from antibiotic-treated (Abx-treated) and control mice. **a** Heatmap of genes involved in the regulation of mitochondria and neurogenesis (the top 30 differentially expressed genes) (Supplementary Tables 4, 5) in corneal tissues of Abx-treated and control mice at postnatal day 28 (P28) (*n* *=* 3 independent experiments, six mice per experiment in each group). **b** The difference in the expression of neurotrophin genes in corneal tissues between Abx-treated mice and control mice with qPCR analysis (*n* *=* 3 independent experiments, six mice per experiment in each group). **c** Comparison of the total length and density of corneal nerve fibers in Abx-treated and control mice at P7, P14, P21, and P28. The two upper images show the staining of nerve fibers with anti-β-III-tubulin antibody in the two groups of mice (scale bars, 500 μm). The two lower graphs show the dynamic changes of the total length and density of the corneal nerve fibers in the two groups of mice at P7, P14, P21, and P28 (*n* *=* 6 mice at each time point in each group). The density of the corneal nerves was obtained through the total length of the corneal nerve fibers dividing the corneal area. The data are presented as mean ± SD. *P < 0.05, **P < 0.01, and ***P < 0.001

Because corneal nerves can secrete neuropeptides to promote mitosis in surrounding epithelial cells,^33–35^ the formation of corneal nerves is also closely associated with corneal morphogenesis. There are many mitochondria within the beads of corneal nerve fibers,^36^ and these mitochondria are critical for the formation of nerve fibers.^37^ RNA-Seq analysis showed that the corneal genes of Abx-treated mice at P28 involved in the regulation of mitochondria and neurogenesis decreased compared with those of control mice (Fig. 3a). A qPCR analysis showed that the expression of the neurotrophin genes *brain-derived neurotrophic factor (Bdnf)*, *neurotrophin (Ntf)-3*, *Ntf-5*, and *nerve growth factor* (*Ngf)*, in corneal tissues were significantly decreased after Abx treatment within 4 postnatal weeks (Fig. 3b). The above results suggest that corneal neurogenesis may have been impaired. The total lengths of corneal nerves in Abx-treated mice were all lower than those in control mice at P14, P21, and P28, and the density of corneal nerves in Abx-treated mice was lower than that in control mice at P14 and P28 (Fig. 3c).

### Oral Abxs do not affect local microbiota on the ocular surface

To investigate whether corneal developmental retardation is related to oral Abx-induced alteration of microbiota on the ocular surface, the microbiota composition profile of the ocular surface was surveyed by 16S rRNA sequencing of normal control and Abx-treated C57BL/6 mice at P21. In both groups, the ocular surface microbiota were mainly composed of *Firmicutes*, *Proteobacteria*, *Bacteroidetes*, and *Actinobacteria* at the phylum level, and *Atopostipes*, *Staphylococcus*, *Stenotrophomonas*, *Streptococcus*, *Pseudomonas*, *Anoxybacillus*, *Rhizobacter*, *Phyllobacterium*, *Rhodococcus*, *Megamonas*, and *Zoogloea* at the genus level (Supplementary Fig. 4a). Further analysis showed no significant difference in the richness or alpha-diversity of gut microbiota between the two groups (Supplementary Fig. 4b, c).

### Fecal transplant promotes the corneal development process in Abx-treated mice

**Fig. 4:**
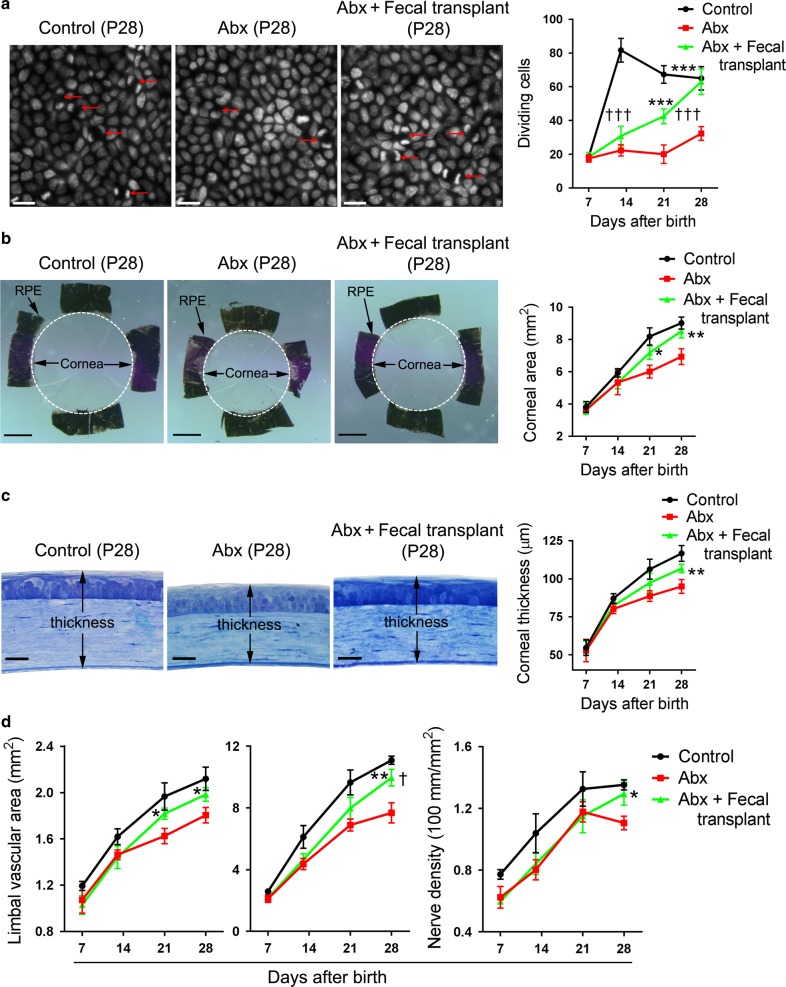
Alteration of corneal development in Abx-treated mice after fecal transplant. **a** Evaluation of the difference in the proliferation capacity among control mice (sterile water treatment), antibiotic-treated (Abx-treated) mice, and Abx-treated mice with fecal transplants at postnatal day 7 (P7), P14, P21, and P28. The three left images show the DAPI staining of corneas from the three groups of mice (scale bars, 25 μm), and the right graph shows the dynamic changes of dividing basal epithelial cells in the three groups of mice after birth (*n* *=* 6 mice at each time point). **b** Evaluation of the difference in the corneal area among control mice, Abx-treated mice, and Abx-treated mice with fecal transplants. The three left images show the spatial range of the cornea as distinguished by the black retinal pigment epithelium (RPE) in the three groups of mice (scale bars, 500 μm). The right graph shows the dynamic changes in corneal area in the three groups of mice after birth (*n* *=* 6 mice at each time point). **c** Evaluation of the difference in corneal thickness among control mice, Abx-treated mice, and Abx-treated mice with fecal transplants. The three left images show the plastic embedding and toluidine blue staining of eye sections in the three groups of mice (scale bars, 20 μm), and the right graph shows the dynamic changes of corneal thickness in the three groups of mice (*n* *=* 6 mice at each time point in each group). **d** The difference in angiogenesis in the limbal blood vessels and neurogenesis in the corneal nerve fibers among control mice, Abx-treated mice, and Abx-treated mice with fecal transplants. The left graph shows the dynamic changes in the area of the limbal blood vessels in the three groups of mice (*n* *=* 6 mice at each time point in each group). The middle and right graphs show the dynamic changes in the total length and density of the corneal nerve fibers in the three groups of mice (*n* *=* 6 mice at each time point in each group). The data are presented as mean ± SD. *Abx-treated mice with fecal transplants vs Abx-treated mice, ^†^Abx-treated mice with fecal transplants vs control mice, *^,†^*P* < 0.05, **P < 0.01 and ***^,†††^*P* < 0.001

Given the crucial roles of gut microbiota in the growth of infants^15,38^ and our conclusions about Abx treatment inducing dysbiosis of the gut microbiota and affecting corneal development in mice, we inferred that the impairment of corneal development in Abx-treated mice may result from the dysbiosis of gut microbiota. To verify this hypothesis, we reconstituted the gut microbiota in Abx-treated mice with transplants of feces from naive mice. After continuous fecal transplants, a 16S rRNA gene sequencing analysis showed that the gut microbiota of mice at P21 were mostly restored (Supplementary Fig. 5). Then we observed changes in corneal morphogenesis and the formation of limbal blood vessels and corneal nerves after the fecal transplants. Our results revealed that the number of dividing epithelial basal cells in Abx-treated mice with fecal transplants increased, and was higher than that in Abx-treated mice without fecal transplants at P21 and P28, and was even equal to the level in control mice at P28 (Fig. 4a). Along with this result, the corneal area at P21 and P28 and the thickness at P28 in Abx-treated mice with fecal transplants increased when compared with those in Abx-treated mice with no transplant, but they were still slightly lower than those of the control mice at P28 (Fig. 4b, c). The area of the limbal blood vessels and the total length and density of the corneal nerves in Abx-treated mice with fecal transplants also increased significantly compared with those in Abx-treated mice with no transplant, although they were slightly lower than those of the control mice at P28 (Fig. 4d).

### CCR2^−^ macrophages participate in corneal development

**Fig. 5:**
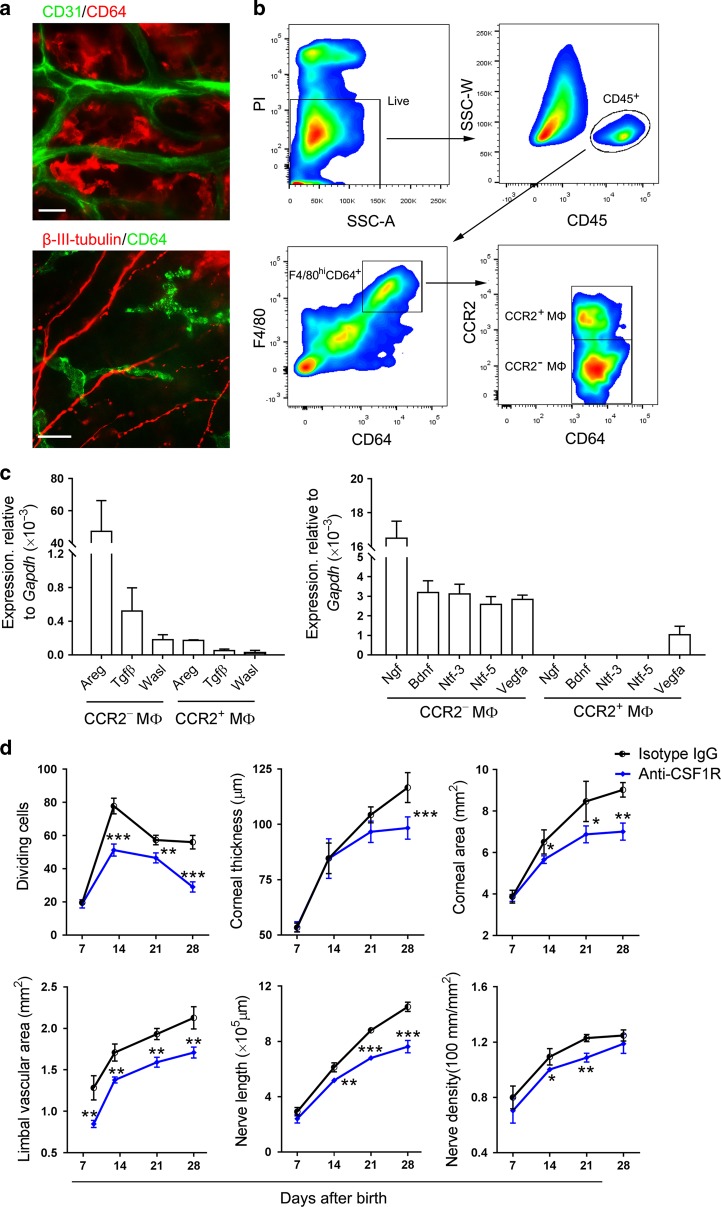

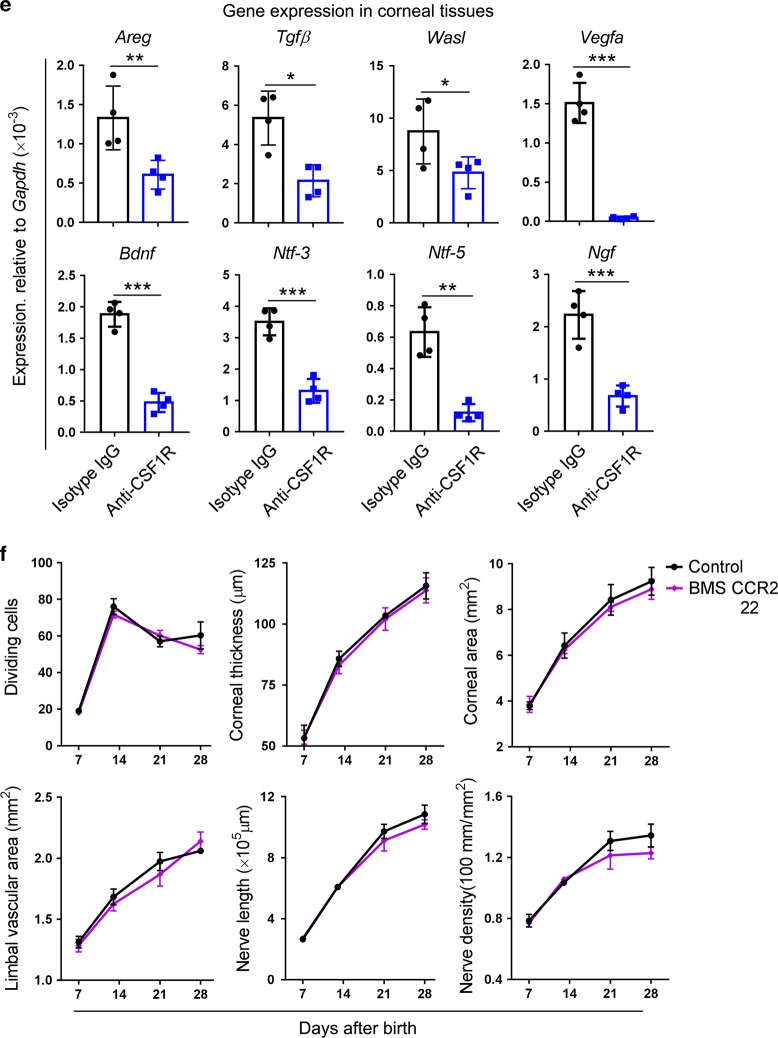
Assessment of the roles of macrophages in corneal development. **a** Co-staining of limbal blood vessels/corneal nerve fibers and macrophages in mice at postnatal day 7 (P7). The upper image shows the immunostaining of the limbal blood vessels and macrophages with anti-CD31 antibody and anti-CD64 antibody (scale bar, 10 μm). The lower image shows the immunostaining of the corneal nerve fibers and macrophages with anti-β-III-tubulin antibody and anti-CD64 antibody (scale bar, 10 μm). **b** The gating strategies of analyzing corneal macrophages with flow cytometry. Corneal macrophages in mice at P7 were classified into C–C chemokine receptor type 2 negative (CCR2^−^) and positive (CCR2^+^) populations. The gating strategies were justified by the related isotype controls in Supplementary Fig. 6. **c** The expression of genes involved in cell division (left image) and neurogenesis and angiogenesis (right image) in the flow-cytometry-sorted corneal CCR2^−^ and CCR2^+^ macrophages with qPCR (*n* *=* 3 independent experiments, ten mice per experiment in each group). **d** Changes in the corneal development of anti-CSF1R antibody-treated mice (*n* *=* 6 mice at each time point in each group). **e** Alteration of the expression of genes involved in cell division, neurogenesis, and angiogenesis in corneal tissues of anti-CSF1R antibody-treated mice at P28 (*n* = 4 independent experiments, six mice per experiment in each group). **f** Changes in the corneal development of BMS CCR2 22-treated mice (*n* *=* 6 mice at each time point in each group). The data are presented as mean ± SD. *P < 0.05, **P < 0.01 and ***P < 0.001

Given the vital roles of macrophages in development,^25–27^ we considered whether these cells participate in corneal development. Immunostaining of limbal blood vessels with anti-CD31 antibody and of macrophages with anti-CD64 antibody revealed that plenty of macrophages are located around limbal blood vessels, and immunostaining of macrophages with anti-CD64 antibody and of corneal nerve fibers with anti-β-III-tubulin antibody showed that the macrophages were also adjacent to the nerve fibers (Fig. 5a). Our previous study indicated that corneal macrophages are classified into C–C chemokine receptor type 2 negative (CCR2^−^) and positive (CCR2^+^) macrophages, and the two cell populations have distinct roles in the regeneration of the corneal epithelium.^39^ In this study, the analysis of gene expression in flow-cytometry-sorted CCR2^−^ and CCR2^+^ corneal macrophages disclosed that both the CCR2^−^ and CCR2^+^ corneal macrophages expressed the *amphiregulin* (*Areg*) gene, a type of epidermal growth factor,^40^ and the *Tgfβ* and *Wasl* genes which were reported to participate in corneal development.^41^ The expression of these genes was significantly lower in the CCR2^+^ corneal macrophages than the CCR2^−^ corneal macrophages (Fig. 5c). CCR2^−^ corneal macrophages expressed the neurotrophic genes *Bdnf*, *Ntf-3*, *Ntf-5*, and *Ngf*, whereas CCR2^+^ corneal macrophages did not; both CCR2^−^ and CCR2^+^ corneal macrophages expressed the blood vessel growth factor gene *Vegfa*, but the expression of *Vegfa* by the CCR2^−^ corneal macrophages was double that of the CCR2^+^ corneal macrophages (Fig. 5c). To observe the function of these cells in corneal development, we used our previous method^39^ to deplete the two macrophage populations. The CCR2^−^ corneal macrophages in the neonatal mice were depleted by the subcutaneous injection of anti-CSF1R antibody, and the CCR2^+^ corneal macrophages were depleted by the subcutaneous injection of the CCR2 antagonist BMS CCR2 22 (Supplementary Fig. 7). The dividing epithelial basal cells, corneal area and thickness, area of limbal blood vessels, and density of corneal nerves in the CCR2^−^ corneal macrophage-depleted mice with an anti-CSF1R antibody injection decreased when compared to those of the Isotype IgG-injected mice (Fig. 5d). Moreover, the expression of *Areg*, *Tgfβ*, *Wasl*, *Vegfa*, *Bdnf*, *Ntf-3*, *Ntf-5*, and *Ngf* in corneal tissues all decreased after anti-CSF1R antibody treatment (Fig. 5e). However, those corneal status showed no significant changes after the CCR2^+^ corneal macrophages were depleted by an injection of the CCR2 antagonist BMS CCR2 22 (Fig. 5f).

### Gut microbiota affect macrophage distribution in the postnatal mouse cornea

**Fig. 6:**
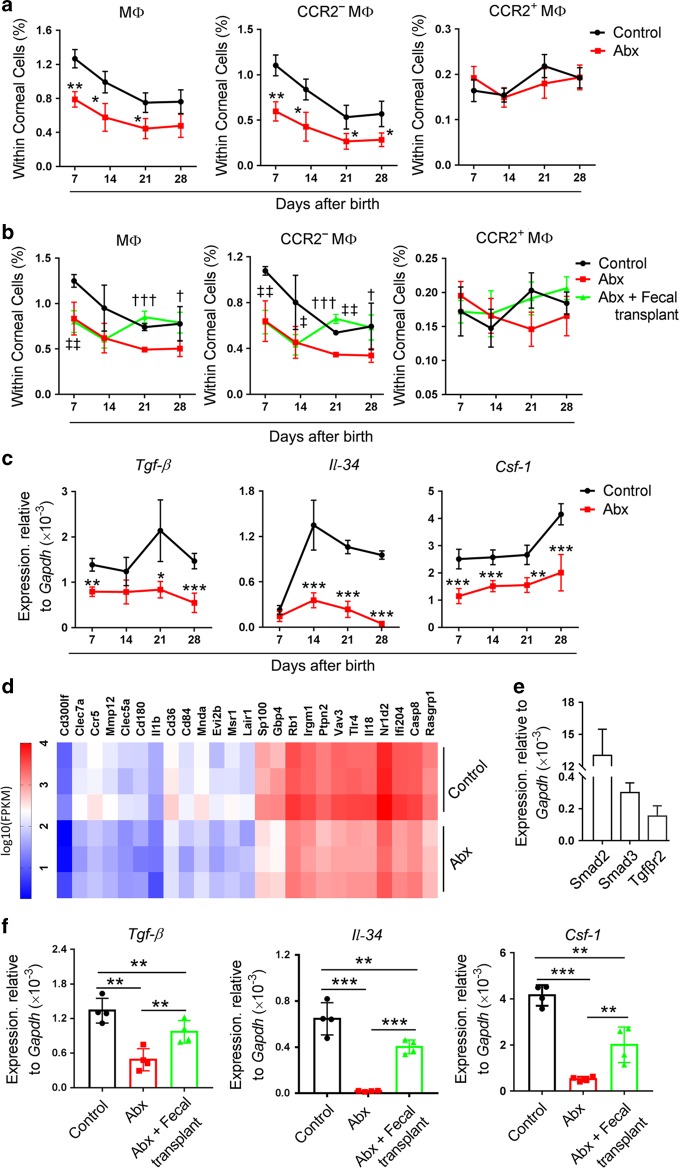
The effects of gut microbiota on the distribution of macrophages in the mouse cornea after birth. **a** Dynamic changes in the proportion of macrophages within corneal cells in antibiotic-treated (Abx-treated) and control mice at postnatal day 7 (P7), P14, P21, and P28 (*n* *=* 3 independent experiments, ten mice per experiment in each group). **b** Changes in the distribution of macrophages within the cornea in Abx-treated mice after fecal transplants (*n* *=* 3 independent experiments, ten mice per experiment in each group). **c** Investigation of the expression of *Tgf-β*, *Il-34*, and *Csf-1* in corneal tissues of Abx-treated and control mice at P7, P14, P21, and P28 (*n* *=* 4 independent experiments, six mice per experiment in each group). **d** Heatmap of genes (top 25 differentially expressed genes) (Supplementary Table 6) involved in the proliferation, survival, and differentiation of macrophages in corneal tissues of Abx-treated and control mice at P28 (*n* *=* 3 independent experiments, six mice per experiment in each group). **e** Analysis of expression of *Smad2*, *Smad3*, and *Tgfβr2* in flow-cytometry-sorted CCR2^−^ corneal macrophages (*n* *=* 3 independent experiments, ten mice per experiment in each group). **f** Investigation of expression of *Tgf-β*, *Il-34*, and *Csf-1* in corneal tissues of control mice, Abx-treated mice, and Abx-treated mice with fecal transplant at P28 (*n* *=* 4 independent experiments, six mice per experiment in each group). The data are presented as mean ± SD. *Abx-treated mice vs control mice, ^†^Abx-treated mice with fecal transplants vs Abx-treated mice, ^‡^Abx-treated mice with fecal transplants vs control mice, *^,‡^*P* < 0.05 and **^,‡‡^*P* < 0.01, ***^,†††^*P* < 0.001

A previous study indicated that the maturation and function of microglia, the tissue-resident macrophages in the CNS, were controlled by the host microbiota.^18^ In this study, we investigated whether the gut microbiota affected the distribution of macrophages in the mouse cornea after birth and found that the proportion of macrophages within corneal cells decreased at P7, P14, and P21 after Abx treatment. Among these macrophages, the proportion of CCR2^−^ macrophages within corneal cells in Abx-treated mice decreased at P7, P14, P21, and P28 when compared with that of control mice, whereas the CCR2^+^ macrophages did not significantly decrease (Fig. 6a). Finally, we observed changes in the macrophages when the gut microbiota in Abx-treated mice were reconstituted through fecal transplants and found that CCR2^−^ macrophages were restored at P21 and P28 after transplants (Fig. 6b).

### Oral abxs change local corneal niches for CCR2^–^ macrophage development

The development and maturation of different tissue-resident macrophages are regulated by different local niche signals and signal-dependent transcription factors.^42^ Our previous study revealed that CCR2^−^ corneal macrophages have a similar gene expression profile and phenotype to microglia and expressed CSF1R, the receptor of IL-34 and CSF1.^39^ To determine the possible molecular association between the development of CCR2^−^ macrophages and niche alteration induced by gut dysbiosis, we compared the expression levels of cytokine transcripts (*Tgf-β*, *Il-34*, and *Csf-1*) from normal control and Abx-treated corneas at different time points (postnatal 7, 14, 21, and 28 days). The data revealed that after Abx treatment, the expression of *Tgf-β*, *Il-34*, and *Csf-1* significantly decreased compared with expression in normal mice (Fig. 6c). Moreover, RNA-Seq analysis showed that 54 genes involved in the proliferation, differentiation, and survival of macrophages decreased after Abx treatment when measured at P28 (Fig. 6d; Supplementary Table 6). We also determined the expression levels of TGF-β receptors and the related transcription factor genes. The qPCR data revealed that CCR2^−^ corneal macrophages expressed *Tgfβr2*, the receptor of *Tgf-β*, and the transcription factor genes *Smad2* and *Smad3* (Fig. 6e). *Smad2* and *Smad3* were also shown to be expressed in microglia.^42^ The expression of *Tgf-β*, *Il-34*, and *Csf-1* in corneal tissues of Abx-treated mice was found to be significantly increased after fecal transplant, although the expression of these genes in corneal tissues of mice with Abx treatment and fecal transplant did not reach the level in normal control mice (Fig. 6f). Altogether, these results confirmed that the local niche for maturation and distribution of CCR2^−^ corneal macrophages was regulated by the gut microbiota.

### Probiotic treatment promotes corneal development in Abx-treated mice

**Fig. 7:**
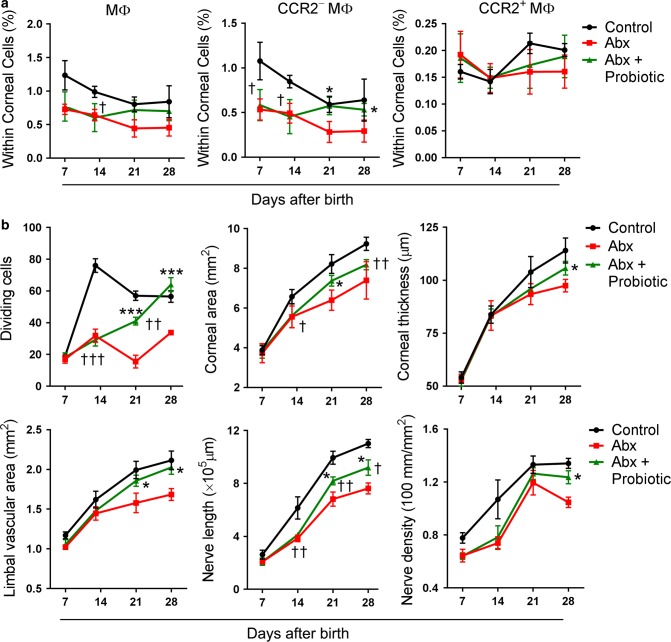
The effects of probiotic treatment on the distribution of macrophages within the cornea and on the corneal development in antibiotic-treated (Abx-treated) mice. **a** Changes in the proportion of macrophages within corneal cells in Abx-treated mice after probiotic transplant at postnatal day 7 (P7), P14, P21, and P28 (*n* *=* 3 independent experiments, ten mice per experiment in each group). **b** Alteration of the corneal development in Abx-treated mice after probiotic transplant at P7, P14, P21, and P28 (*n* *=* 6 mice at each time point in each group). The data are presented as mean ± SD. *Abx-treated mice with probiotic transplant vs Abx-treated mice, ^†^Abx-treated mice with probiotic transplant vs control mice, *^,†^*P* < 0.05, ^††^*P* < 0.01 and ***^,†††^*P* < 0.001

Probiotics are live microorganisms that can provide health benefits to the host when consumed in adequate amounts.^43,44^ VSL#3 is a probiotic mixture containing eight bacterial strains that have been reported to restore hippocampal neurogenesis and brain function in Abx-treated mice.^17^ Thus, we decided to give a VSL#3 probiotic mixture to Abx-treated mice and observed the changes in corneal development. CCR2^−^ corneal macrophages in Abx-treated mice increased at P21 and P28 after the probiotic transplant (Fig. 7a). In addition, epithelial basal cell division in Abx-treated mice with the probiotic transplant increased at P21 and P28, and was even slightly higher than that in control mice at P28. The corneal area and thickness, limbal blood vessel area, and the total length and density of corneal nerves in Abx-treated mice with the probiotic transplant were also increased, but they were still lower than those of the control mice at P28 (Fig. 7b).

### Long-term consequences of early Abx treatment for corneal status

**Fig. 8:**
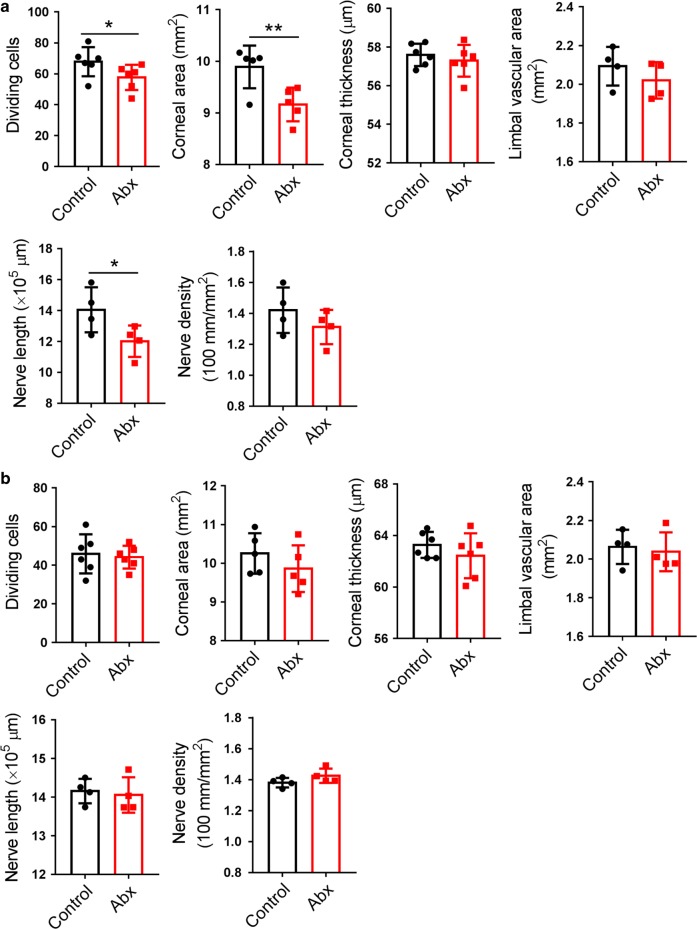
The long-term effects of early Abx treatment on corneal status. **a** Comparison of corneal epithelial dividing cell, corneal area and thickness, limbal vascular area, and length and density of corneal nerve fibers between Abx-treated mice and control mice at postnatal 8 weeks (*n* *=* 6 mice in each group). **b** Comparison of corneal epithelial dividing cell, corneal area and thickness, limbal vascular area, and length and density of corneal nerve fibers between Abx-treated mice and control mice at postnatal 12 weeks (*n* *=* 6 mice in each group). *P < 0.05, **P < 0.01

Our data showed that the decreases in corneal epithelial dividing cell number, area and thickness (Fig. 1), limbal blood vessel area (Fig. 2), and corneal nerve fiber length and density (Fig. 3) were still visible at P28 after Abx treatment finished at P14. To investigate the long-term consequences of early Abxs for corneal status, we continued to observe the corneal status of Abx-treated mice at postnatal 8 and 12 weeks and found that the corneal dividing cell number, the corneal area, and the corneal nerve fiber length in Abx-treated mice were still lower than those in control mice at postnatal 8 weeks (Fig. 8a). However, none of the phenotypes observed above showed any significant difference between the control mice and the Abx-treated mice at postnatal 12 weeks (Fig. 8b). We further analyzed the composition of the gut microbiota and found that at postnatal 8 weeks, the gut microbiota in Abx-treated mice had recolonized and were not significantly different from those in control mice (Supplementary Fig. 8).

## Discussion

Although Abxs are remarkably useful, these drugs also have adverse impacts on the host. In this study, we found that the continuous use of Abxs caused dysbiosis of the gut microbiota in neonatal mice and impaired their corneal development through the alteration of the corneal gene transcription, and decreases in the corneal area and thickness, the area of limbal blood vessels, the length and density of corneal nerve fibers, and the proportion of CCR2^−^ corneal macrophages. With the reconstitution of gut microbiota by fecal transplants, the deficiency in corneal development in Abx-treated mice could mostly be reversed. Further investigation confirmed that CCR2^−^ corneal macrophages were crucial for corneal development and that gut microbiota affected the distribution of these cells in the cornea. Moreover, the application of VSL#3 probiotics was shown to promote corneal development in Abx-treated mice.

The use of Abxs has negative impacts on the host’s homeostasis and physiology, causing a decrease in nitrergic neurons in the intestine,^45^ the delay of gastrointestinal peristalsis,^45^ the impairment of hippocampal neurogenesis and memory retention,^17^ and defects in hematopoiesis.^46,47^ In our study, long-term Abx treatment was found to change the composition of the gut microbiota and the transcription of corneal genes and to decrease the abundance of genes involved in corneal development. Furthermore, the area and thickness of the cornea, the area of the limbal blood vessels, and the length and density of the corneal nerve fibers in Abx-treated mice were all lower than those in the control mice. It has been reported that there are two possible reasons for Abx-induced changes in the intestinal tissues: the Abx-induced dysbiosis of gut microbiota and the direct toxic effect of Abxs.^48^ After the reconstitution of gut microbiota through fecal transplants, the deficiency in the corneal development of Abx-treated mice could mostly be reversed. Therefore, it can be argued that the dysbiosis of gut microbiota caused by Abx treatment is a primary factor that impairs corneal development, although we do not exclude the possibility of the toxic effects of Abxs on corneal development. It is consistent with the results of Wang et al. that gut microbiota-deficient mice, germ-free mice, which has abnormal cornea, performance in the disruption and increased permeability of corneal barrier.^49^

Studies have shown that the gut microbiome composition in mice receiving Abxs is not reestablished even 6 weeks after the cessation of the drug.^50,51^ Studies in human infants have shown that a full recovery of the intestinal flora after Abx use takes months.^52,53^ In this study, we found that the gut microbiome in Abx-treated mice was recolonized in composition and diversity at 6 weeks after the end of Abx treatment. However, the characteristics of the mouse cornea had still not returned to normal in that time. It was not until 10 weeks after the withdrawal of the drug that the corneal development reached normal levels. These results suggest that Abx-induced gut dysbiosis has significant, long-term effects on the development of the cornea. Although this suppressive effect is reversible, the reversal takes a long time.

Macrophages are innate immune cells that have been reported to participate in the development of many tissues and organs. For instance, the investigation of PU.1^−/−^, Csf1^op/op^, and Csf1r^−/−^ mice, which lack many macrophage populations, has revealed a cluster of developmental abnormalities: tooth eruption and osteopetrosis in Csf1^op/op^ and Csf1r^−/−^ mice,^29,30^ remodeling deficiencies of the mammary gland, kidney, and pancreas in Csf1r^−/−^ mice,^54,55^ a failure of remodeling and persistence of the vascular structure in PU.1-deficient mice,^56^ and neuronal defects of the brain in Csf1^op/op^ mice.^57^ Macrophages are categorized as M1 and M2 subtypes according to their different cytokine secretion and function.^58–61^ In the formation of blood vessels and nerve fibers, M2 macrophages are more powerful in promoting angiogenesis and neurogenesis when compared with M1 macrophages.^62,63^ In this study, we confirmed that depletion of corneal CCR2^−^ macrophages decreased the area and thickness of the cornea, the area of the limbal blood vessels, and the length and density of the corneal nerve fibers. Thus, corneal CCR2^−^ macrophages are similar to M2 macrophages regarding their roles in the development process and participate in postnatal morphogenesis of the cornea and the formation of the limbal blood vessels and corneal nerves.

Gut microbiota have been reported to influence the migration, distribution, and function of various immune cells.^64^ Erny et al. found that symbiotic bacteria in the gastrointestinal tract regulated the distribution and function of microglia in the brain, and the impairment of immune responses induced by a deficiency of gut microbiota can be reversed to some extent by the transplant of complex microbiota.^18^ In our study, the proportion of CCR2^−^ macrophages within corneal cells in Abx-treated mice was significantly lower than that of the control mice, and could be reversed by the reconstitution of gut microbiota through fecal transplants.

The development and maturation of brain microglia depend on local niche signals, such as TGF-β, IL-34, and CSF1 cytokines, and signal-dependent transcription factors, such as SALL1 and SMAD2/3.^42^ Similarly, we found that the expression of *Tgf-β*, *Il-34*, and *Csf-1* and some other genes involved in the proliferation, survival, and differentiation of macrophages in corneal tissues all decreased. Also, the expression of *Tgf-β*, *Il-34*, and *Csf-1* in the corneal tissues of Abx-treated mice can be partially reversed with fecal transplants. Moreover, CCR2^−^ corneal macrophages were shown to express the *Tgf-β* receptor *Tgfβr2*, and the *Il-34* and *Csf-1* receptor *Csf-1r*, and the transcription factor genes *Smad2* and *Smad3*. These findings indicate that the distribution of CCR2^−^ corneal macrophages acts in a manner similar to microglia that are controlled by gut microbiota by affecting the expression of local cytokines.

It is becoming increasingly clear that supplementing with certain microbial strains is beneficial for our health.^65^ VSL#3 is a commercial probiotic mixture containing eight bacterial strains. It has been reported that these bacterial strains can alleviate the inflammatory responses in inflammatory bowel disease.^66^ After dysbiosis of the gut microbiota induced by Abx treatment, hippocampal neurogenesis and memory function decrease, but a VSL#3 transplant can reverse the impaired performance in the hippocampus.^17^ In this study, we also saw consistent results showing that a transplant of the VSL#3 probiotic mixture is beneficial for reversing the impairment of corneal development in Abx-treated mice and restoring the proportion of CCR2^−^ macrophages. Thus, our results confirm the potential for probiotics to be a therapeutic strategy for curing a deficiency in corneal development induced by dysbiosis of gut microbiota.

Collectively, our results suggest that Abx treatment causes dysbiosis of gut microbiota and impairs corneal development, including by altering corneal morphogenesis and the formation of the limbal blood vessels and corneal nerves. These deficiencies in corneal development can mostly be reversed by the reconstitution of gut microbiota through fecal transplants and by the probiotic treatment. In addition, CCR2^−^ macrophages have been confirmed to participate in corneal development, and the distribution of these cells is regulated by gut microbiota. Although the deficiency in corneal development induced by Abx treatment is an intricate process, our findings illuminate the important roles of homeostatic gut microbiota in corneal development and highlight the importance of CCR2^−^ macrophages in this development process.

## Materials and methods

### Animals

Specific Pathogen-Free (SPF) female C57BL/6 mice without eye diseases, aged P0–P90, were fed in the Animal Center at Jinan University. All animal protocols in this study were approved by the Jinan University Laboratory Animal Committee on Animal Welfare. All the animals were treated in accordance with the Association for Research in Vision and Ophthalmology’s Statement for the Use of Animals in Ophthalmology and Vision Research and the guidelines of the Animal Experimental Committee at Jinan University. The animals were anesthetized by inhalation of 2% isoflurane and euthanized by an overdose of CO_2_ and cervical dislocation. To avoid the effects of circadian rhythms to dividing epithelial cells, all corneal samples were obtained at the same time.

### Antibiotic treatment

Neonatal mice at postnatal 1–7 days and postnatal 8–14 days were separately provided 10 μl and 30 μl of an aqueous solution of antibiotic cocktail, including ampicillin (1 g/L; Sigma-Aldrich, St. Louis, MO; no. A1593), vancomycin (500 mg/L; Sigma-Aldrich; no. V2002), neomycin sulfate (1 g/L; Sigma-Aldrich; no. N0401000), and metronidazole (1 g/L; Sigma-Aldrich; no. M3761) each day by gavage with a stainless steel mouse gavage needle (1-inch, 24-gauge, 1.25-mm ball needle; Harvard Apparatus, Holliston, MA). The control mice were given sterile water.

### 16S rRNA gene sequencing

The species and abundances of the gut microbiota were analyzed by 16S rRNA gene sequencing. The intestinal tissues and the contents of these gut tracts were collected to extract genomic DNA with the QIAamp DNA Stool Mini Kit, and this genomic DNA were sent to the TinyGene company (Shanghai, China) for 16S rRNA gene sequencing. The intestinal tissues and gut contents of one mouse were obtained as one experimental sample, and six independent experiments were carried out.

### Fecal transplant and probiotic administration

Neonatal mice were orally administrated the cocktail of antibiotics for 14 consecutive days, and were then given sterile water for 2 days. After that, these mice were divided into two groups: one group continually treated with sterile water, and the other group orally treated with fecal flora (the feces of five mice were mixed and given to ten mice) or with unflavored VSL#3 probiotic mixture (VSL Pharmaceuticals Inc., Towson, MD, USA) each day. The method for preparing fecal flora was referred to in our previous study.^67^ Donor mice, the same age as the recipient mice, were chosen. The intestines and their contents were collected from the donor mice, cut into pieces, and washed in saline. Then, the mixture was passed through a 75-μm filter to obtain the suspension of gut microorganisms. This suspension of gut microorganisms from five mice was then diluted with saline to 500 μl and given to ten mice. All the operations were performed in a sterile environment. VSL#3 is a commercial probiotic cocktail containing eight bacterial strains: *Bifidobacterium breve*, *Bifidobacterium longum*, *Bifidobacterium infantis*, *Lactobacillus acidophilus*, *Lactobacillus plantarum*, *Lactobacillus paracasei*, *Lactobacillus bulgaricus*, and *Streptococcus thermophiles*. Each mouse was given nearly 1 × 10^7^ probiotic bacteria orally.

### RNA-Seq for transcriptional analysis

After the mice were euthanized, their eyeballs were obtained and clipped under a dissecting microscope in phosphate buffered solution (PBS) to maintain a complete cornea. These corneas were then cut into pieces, placed into Buffer RZ (Tiangen, Beijing, China; no. RK145), and broken up with a TissueRuptor (Qiagen, Germantown, MD). After that, the total RNA of the corneal tissues were extracted with an RNAsimple Total RNA Kit (Tiangen; no. DP419) and sent to the BGI company (Guangdong, China) for mRNA sequencing. For the differentially expressed genes, a volcano plot was made using Excel software (Microsoft, Redmond, USA). A gene ontology analysis was carried out with the Gene Ontology Consortium. The target genes were sorted from the differentially expressed genes according to the certain biological process. These genes were then used to make heat maps with GraphPad Prism 7 (GraphPad Software, La Jolla, USA). Twelve corneas from six mice were pooled as one experimental sample, and three independent experiments were carried out in the RNA-Seq analysis.

### Macrophage depletion

The method for the depletion of macrophages is referred to in our previous study.^39^ Neonatal mice were subcutaneously injected with anti-CSF1R antibody (0.5 µg/µl; eBioscience, San Diego, CA; no. 16-1152-82) once every other day for a total of five times to deplete the CCR2^−^ macrophages. Each mouse, at postnatal 1–5 days, was subcutaneously injected with 10 μl of the anti-CSF1R antibody; at postnatal 6–10 days, each mouse was subcutaneously injected with 20 μl of the anti-CSF1R antibody. Control mice were subcutaneously injected with an equal amount of Isotype IgG. In addition, newborn mice were subcutaneously injected with the CCR2 antagonist BMS CCR2 22 (10 μg/μl) once every other day for a total of five times to deplete the CCR2^+^ macrophages. The amount of BMS CCR2 22 given was based on the weight of each mouse (0.5 mg/kg). Control mice were subcutaneously injected with an equal amount of the vehicle for BMS CCR2 22. BMS CCR2 22 (R&D Systems, Minneapolis, MN; no. 3129) was dissolved in ethanol and then diluted with saline to the appropriate concentration.

### Immunostaining and qualitative analysis

This analysis was performed as in our previous studies.^68–73^ Briefly, after euthanasia, the eyeballs of the mice were fixed in 4% paraformaldehyde for 1 h and then clipped in PBS under a dissecting microscope to maintain the corneas with complete limbi. These corneas were blocked in 2% bovine serum albumin (BSA) for 15 min, permeabilized with 0.1% Triton X-100/2% BSA for 15 min and incubated overnight at 4 °C in a mixture of the following antibodies: anti-mouse CD31-FITC (1:100; BD Biosciences, San Jose, CA; no. 553372), anti-mouse CD64-PE (1:100; BioLegend, San Diego, CA; no. 139303) and anti-β-III-tubulin conjugated with NL637 (1:10; R&D Systems; no. NL1195R). After that, the corneal tissues were washed in PBS three times (5 min each time), then placed on glass slides, and cut radially to flatten them. A fluorescent mounting medium containing 1 μM 4′,6-diamidino-2-phenylindole (DAPI) (Sigma-Aldrich; no. 28718-90-3) was placed on the corneas. Image analysis of the corneas was performed with a DeltaVision Elite high-resolution microscope (GE Healthcare Life Sciences, Pittsburgh, PA, USA).

Calculation of the limbal blood vessel area was referred to in our previous study.^9^ Images of limbal blood vessels were loaded into Photoshop CS4 software (Adobe company, San Jose, USA). Colored blood vessels were captured, measured, and represented by the total number of pixels. The actual area of limbal blood vessels was calculated by converting the pixels to the original scale of the image. In addition, the total length of the corneal nerve fibers was analyzed by the Imaris software (Bitplane, Switzerland). Briefly, the image of whole cornea was loaded into Imaris software. β-III-tubulin positive nerve fibers could be detected, and their total length was calculated automatically. The corneal area was analyzed with Photoshop CS4 software. Then the density of the corneal nerves was obtained through the total length of the corneal nerve fibers dividing the corneal area. Twelve corneas from six mice were obtained to count the number of dividing basal epithelial cells and to calculate the area and thickness of the cornea, the area of the limbal blood vessels, and the length and density of the corneal nerve fibers.

### Plastic embedding and toluidine blue O staining

This analysis was carried out as in our previous study.^9,69^ Briefly, enucleated eyeballs were fixed overnight in 2.5% glutaraldehyde. The excised corneas were washed and then fixed in 1% osmic acid. After washing with PBS, these tissues were dehydrated and placed in a 1:1 mixture of 100% ethanol:Epon 812 for 3 h and rinsed three times using Epon 812. Semithin corneal sections were obtained with a microtome and stained with 0.4% (w/v) toluidine blue O dissolved in 60% alcohol. The central corneal thickness was calibrated with the built-in imaging software SoftWorx under a DeltaVision Elite system.

### Flow cytometric analysis

Corneal tissues were cut into pieces, and digested in 0.4% collagenase at 37°C for 30 min. After digestion, the corneal cells were washed in PBS, passed through a 75-μm filter, and then blocked in the Flow Cytometry Staining Buffer containing anti-mouse CD16/32 antibody (1:100) at room temperature for 10 min. Furthermore, these cells were incubated at room temperature for 30 min in a mixture of the following antibodies (all 1:100): anti-mouse CD45 antibody conjugated with FITC (BD Biosciences, San Jose, CA; no. 553080), anti-mouse F4/80 conjugated with PerCP- Cyanine5.5 (BioLegend, San Diego, CA; no. 123128), anti-mouse CD64 conjugated with Brilliant Violet 421 (BioLegend, San Diego, CA; no. 139309), and anti-mouse CCR2 conjugated with APC (R&D Systems, no. FAB5538A). After that, these cells were incubated with propidium iodide (PI; Sigma-Aldrich; no. 25535-16-4) for 10 min to exclude dead cells; they were then analyzed using a BD FACSCanto. The gate strategies were justified by related isotype control antibodies: FITC Rat IgG2b, κ Isotype Control (BD Biosciences, San Jose, CA; no. 553988); PerCP-Cy5.5 Rat IgG2a, κ Isotype Control (BioLegend, San Diego, CA; no. 400532); Brilliant Violet 421™ Mouse IgG1, κ Isotype Control (BioLegend, San Diego, CA; no. 400157); Rat IgG2B APC‑conjugated Isotype Control (R&D Systems, no. IC013A). Twenty corneas from 10 mice were pooled as one experimental sample, and three independent experiments were carried out on this sample in the flow cytometric analysis.

### Transcript amplification from corneal macrophages

After being stained with anti-mouse CD45-FITC, CD64-PE, and CCR2-APC antibodies, the corneal cells were analyzed and sorted by a BD FACSAria to obtain the CCR2^−/+^ macrophages. The transcript of the sorted CCR2^−/+^ macrophages was amplified with a REPLI-gWTA Single Cell Kit (Qiagen, no. 150063). Twenty corneas from ten mice were pooled as one experimental sample, and three independent experiments were carried out on this sample.

### qPCR

The corneal tissues were cut into pieces, put into Buffer RZ (Tiangen, no. RK145), and broken up with a TissueRuptor (Qiagen, Germany). With the RNAsimple Total RNA Kit (Tiangen, no. DP419), the total RNA was obtained from the corneal tissues, and treated with a ReverTra Ace qPCR RT Kit (Toyobo, Osaka, Japan; no. FSQ-101) to obtain cDNA. Finally, the expression of the target genes in the cDNA of corneal tissues was executed in a THUNDERBIRD SYBR qPCR Mix (Toyobo; no. QPS-201). Twelve corneas from six mice were pooled as one experimental sample, and three independent experiments were carried out on this sample in the qPCR analysis. The qPCR primers used in this study are shown in Table 1.

**Table 1:** PCR primers used in this study

Gene name	Primer sequence	
*Vegfa*	Forward	5′-GCTCTTCTCGCTCCGTAGTA-3′
	Reverse	5′-CCTCTCCTCTTCCTTCTCTTCC-3′
*Hif1-α*	Forward	5′-AACCACCCATGACGTGCTTG-3′
	Reverse	5′-AGTTCTTCCGGCTCATAACCC-3′
*Nus 1*	Forward	5′-GCCCAGGATTTTTGCCAGTT-3′
	Reverse	5′-GGATCAGGGAACCCATGTGA-3′
*Pdcd10*	Forward	5′-AGGCACGGGCACTTAAACA-3′
	Reverse	5′-CCTGCGGTTCTGGTACTGATAT-3′
*Bdnf*	Forward	5′-GACGGTCACAGTCCTAGAGAA-3′
	Reverse	5′-CCTTATGAATCGCCAGCCAAT-3′
*Ntf-3*	Forward	5′-TACGGCAACAGAGACGCTAC-3′
	Reverse	5′-GTGGTGAGGTTCTATTGGCTAC-3′
*Ntf-5*	Forward	5′-AGGCACTGGCTCTCAGAATG-3′
	Reverse	5′-AGCTGTGTCGATCCGAATCC-3′
*Ngf*	Forward	5′-ACTCATACTGCACCACGACT-3′
	Reverse	5′-TCAGCCTCTTCTTGTAGCCTT-3′
*Areg*	Forward	5′-TTGGTGAACGGTGTGGAGAA-3′
	Reverse	5′-GCGAGGATGATGGCAGAGA-3′
*Tgfβ*	Forward	5′-ACCGCAACAACGCCATCT-3′
	Reverse	5′-ACCAAGGTAACGCCAGGAAT-3′
*Wasl*	Forward	5′-CTTCTCCTTCCTCGGCAAGA-3′
	Reverse	5′-CCTTAACCAGACAAGCGACAC-3′
*Il-34*	Forward	5′-TGACCTTACAGGCTACCTTCG-3′
	Reverse	5′-TCCAGCAATGTCTGAACCTCC-3′
*Csf-1*	Forward	5′-TGAACAGCTGCTTCACCAAGGAC-3′
	Reverse	5′-TCAGGCTTGGTCACCACATCTCG-3′
*Smad2*	Forward	5′-CGTCCATCTTGCCATTCACTC-3′
	Reverse	5′-TCTTCCTGTCCATTCTGCTCTC-3′
*Smad3*	Forward	5′-CCTGGCTACCTGAGTGAAGAT-3′
	Reverse	5′-TGTGAGGCGTGGAATGTCT-3′
*Tgfβr2*	Forward	5′-TTAACAGTGATGTCATGGCCAGCG-3′
	Reverse	5′-AGACTTCATGCGGCTTCTCACAGA-3′
*GAPDH*	Forward	5′-CAAGGACACTGAGCAAGAG-3′
	Reverse	5′-TGCAGCGAACTTTATTGATG-3′

### Statistical analysis

The results are presented as mean ± SD. For comparisons between groups, ANOVA was performed, followed by Tukey’s HSD test. Statistical significance was set at *P* < 0.05.

## Supplementary information

Supplementary information
