# Supplementary material for: Antibiotic-induced dysbiosis of gut microbiota impairs corneal development in postnatal mice by affecting CCR2 negative macrophage distribution
Source: Mucosal Immunol. 2019 Aug 21;13(1):47–63. doi: 10.1038/s41385-019-0193-x (PMC6914671; doi:10.1038/s41385-019-0193-x)
Supplement: Supplementary file 1 — Supplementary information [file 41385_2019_193_MOESM1_ESM.pdf]

Antibiotic-induced Dysbiosis of Gut Microbiota Impairs Corneal Development in Postnatal Mice by Affecting CCR2 Negative Macrophage Distribution

Running title: Gut Microbiota and Corneal Development

Mingjuan Wu[1#], Jun Liu[1#], Fanying Li[1,2], Shuoya Huang[1,3], Jingxin He[1,3], Yunxia Xue[1], Ting Fu[1], Shanshan Feng[1], Zhijie Li[1, 3, 4*]

[1]International Ocular Surface Research Center, Institute of Ophthalmology, and Key Laboratory for Regenerative Medicine, Jinan University, Guangzhou, China

[2]Department of Microbiology and Immunology, School of Medicine, Jinan University, Guangzhou, China;

[3]Department of Ophthalmology, The First Affiliated Hospital of Jinan University, Guangzhou, China;

[4]Department of Ophthalmology, Henan Provincial People's Hospital, Zhengzhou, China

[#]These authors contributed equally to this work and should be considered co-first authors

[*] Corresponding author; email address: tzhijieli@jnu.edu.cn

Supplementary information

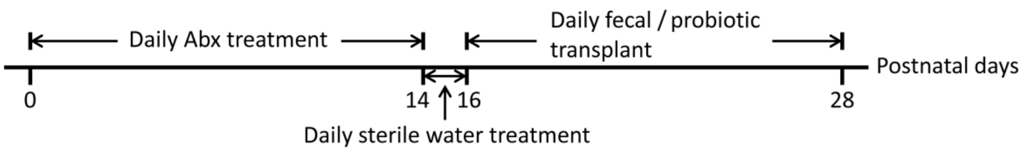

Supplementary Figure 1. Schematic view of the timing of antibiotic (Abx) treatment and fecal /probiotic transplant.

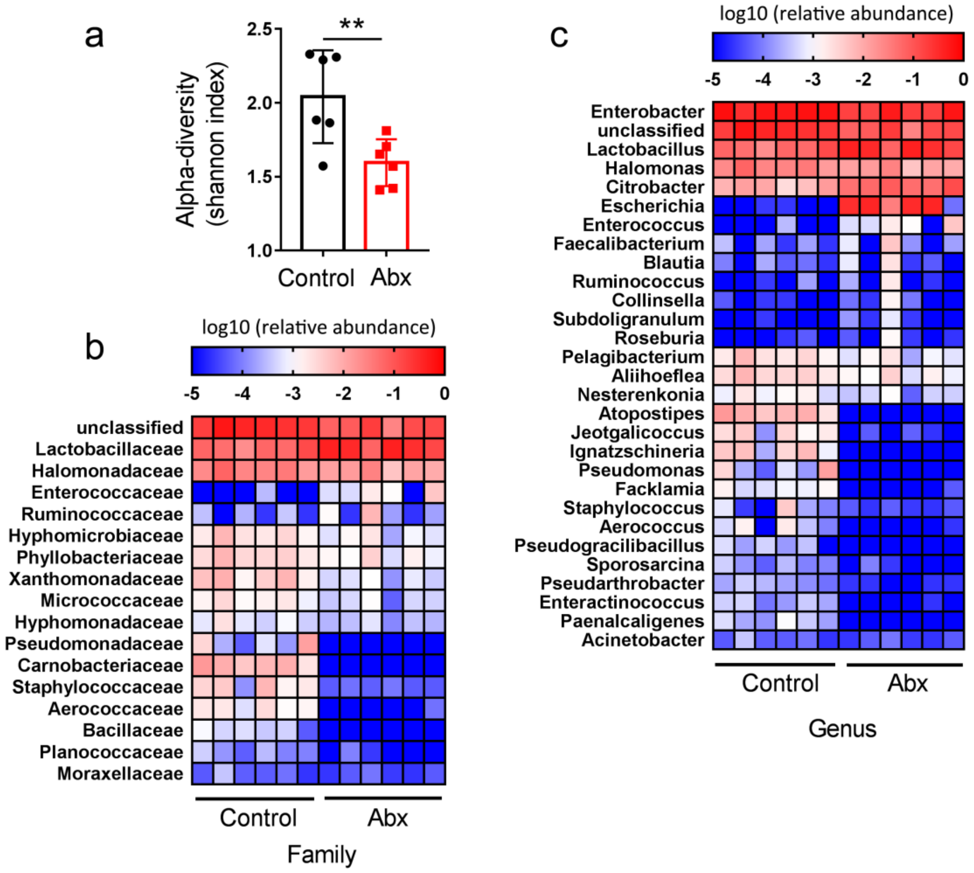

Supplementary figure 2. Investigation of the diversity and abundance of gut microbiota in control and antibiotic-treated mice at P14 with 16S rRNA sequencing. (**a**) Shannon indices of gut microbiota in control and Abx-treated groups (*n* = 6 mice in each group). Gut microbiota of Abx-treated mice have lower Shannon indices than those of control mice. **P < 0.01. (**b**) Heat map of gut microbiota at the family level in the two groups of mice (*n* = 6 mice in each group). (**c**) Heat map of gut microbiota at the genus level in the two groups of mice (*n* = 6 mice in each group).

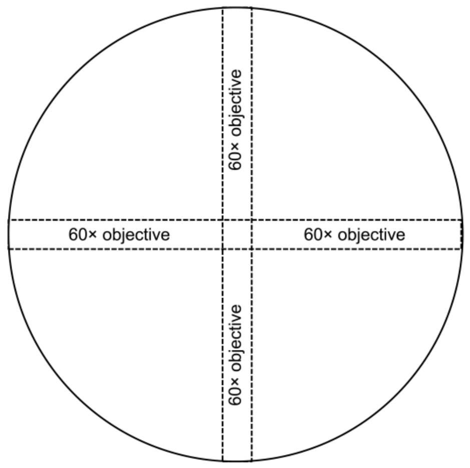

Supplementary figure 3. The schematic view of the cornea with fields in four quadrants (60×

objective). Total number of dividing epithelial basal cells within these fields represents the proliferation capacity of corneal epithelial cells.

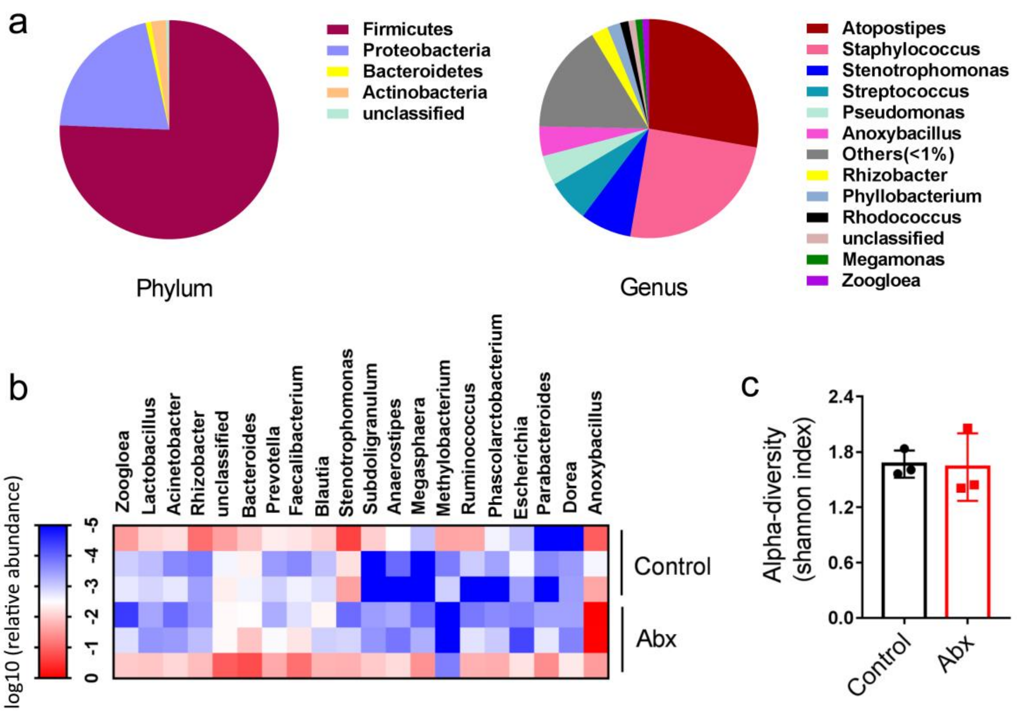

Supplementary Figure 4. Investigation of the diversity and abundance of local microbiota on the ocular surfaces of control and antibiotic-treated mice at P21 with 16S rRNA sequencing. (**a**) The composition of ocular microbiota at the phylum and genus levels. (**b**) Heat map of ocular microbiota at the genus level in the two groups of mice (*n* = 3 mice in each group). (**c**) Shannon indices of ocular microbiota in the control and Abx-treated groups (*n* = 3 mice in each group).

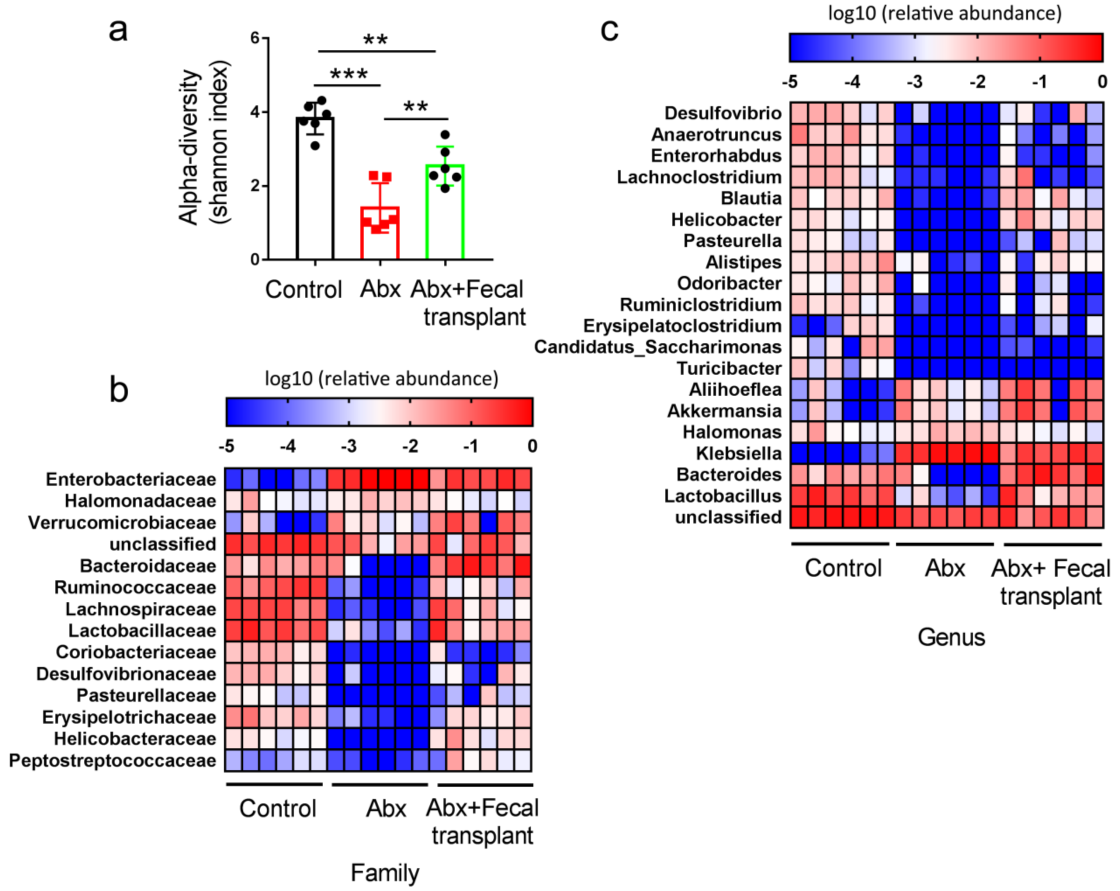

Supplementary figure 5. Investigation of the diversity and abundance of gut microbiota in control mice, antibiotic-treated mice and antibiotic-treated mice with fecal transplant at P21 with 16S rRNA sequencing. (**a**) Shannon indices of gut microbiota in control mice, Abx-treated mice and Abx-treated mice with fecal transplant (*n* = 6 mice in each group). **P < 0.01, ***P < 0.001. (**b**) Heat map of gut microbiota at the family level in the three groups of mice (*n* = 6 mice in each group). (**c**) Heat map of gut microbiota at the genus level in the three groups of mice (*n* = 6 mice in each group).

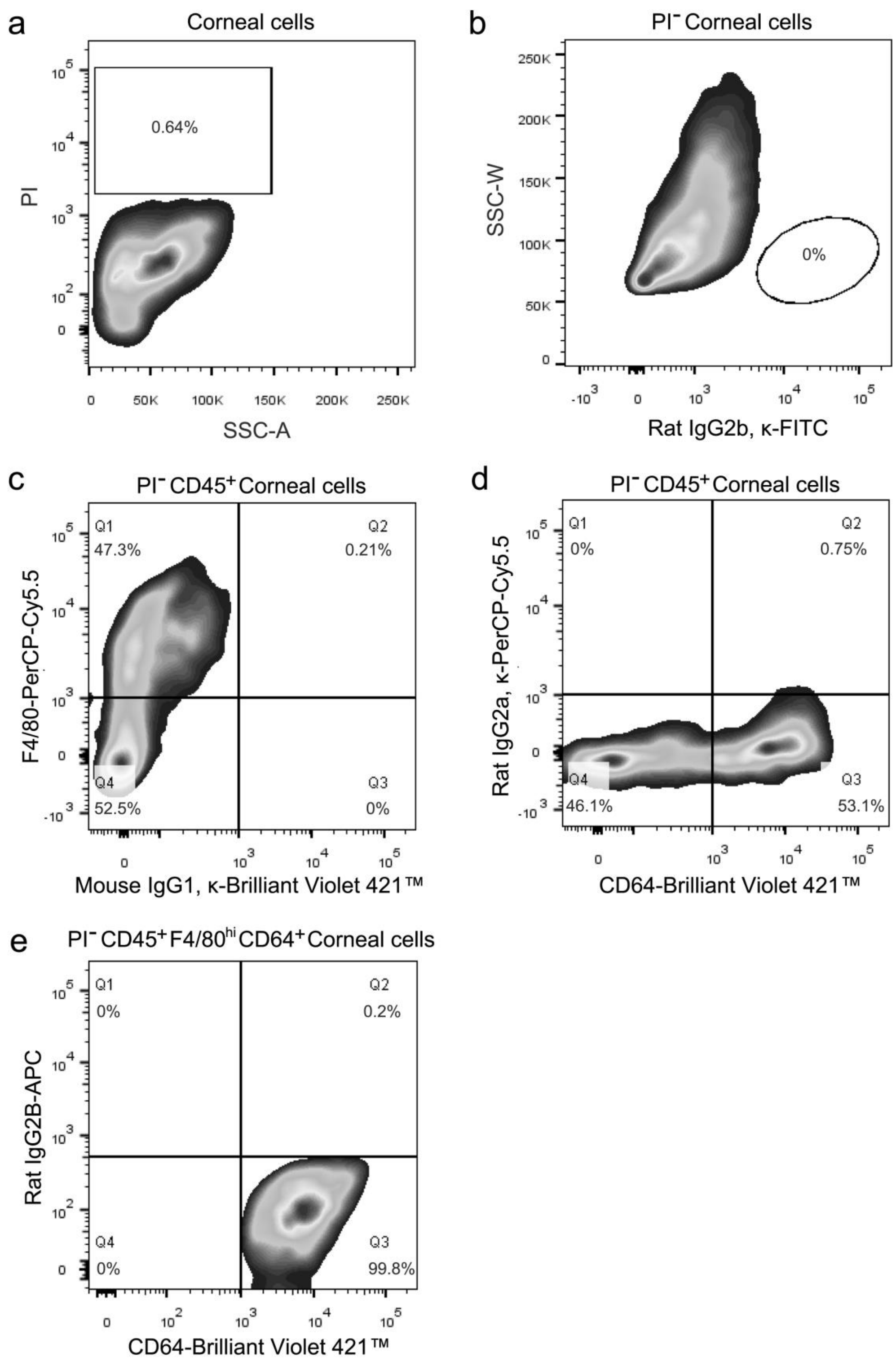

Supplementary figure 6. Isotype controls for justifying the gating strategies used in the panel B of figure 5, using the fluorescence-minus-one (FMO) gate method. (**a**) The gate strategy for propidium iodide (PI) staining with anti-mouse CD45 FITC-conjugated antibody, anti-mouse F4/80 PerCP-Cy5.5-conjugated antibody, anti-mouse CD64 Brilliant Violet 421-conjugated antibody, and anti-mouse CCR2 APC-conjugated antibody stained corneal cells. (**b**) The gate strategy for

anti-mouse CD45 FITC-conjugated antibody staining with PI, Rat IgG2b, κ-FITC, anti-mouse F4/80 PerCP-Cy5.5-conjugated antibody, anti-mouse CD64 Brilliant Violet 421-conjugated antibody, and anti-mouse CCR2 APC-conjugated antibody stained corneal cells. (**c**) The gate strategy for anti-mouse CD64 Brilliant Violet 421-conjugated antibody staining with PI, anti-mouse CD45 FITC-conjugated antibody, anti-mouse F4/80 PerCP-Cy5.5-conjugated antibody, Mouse IgG1, κ-Brilliant Violet 421™, and anti-mouse CCR2 APC-conjugated antibody stained corneal cells. (**d**) The gate strategy for anti-mouse F4/80 PerCP-Cy5.5-conjugated antibody staining with PI, anti-mouse CD45 FITC-conjugated antibody, Rat IgG2a, κ-PerCP-Cy5.5, anti-mouse CD64 Brilliant Violet 421-conjugated antibody, and anti-mouse CCR2 APC-conjugated antibody stained corneal cells. (**e**) The gate strategy for anti-mouse CCR2 APC-conjugated antibody staining with PI, anti-mouse CD45 FITC-conjugated antibody, anti-mouse F4/80 PerCP-Cy5.5-conjugated antibody, anti-mouse CD64 Brilliant Violet 421-conjugated antibody, and Rat IgG2B–APC stained corneal cells.

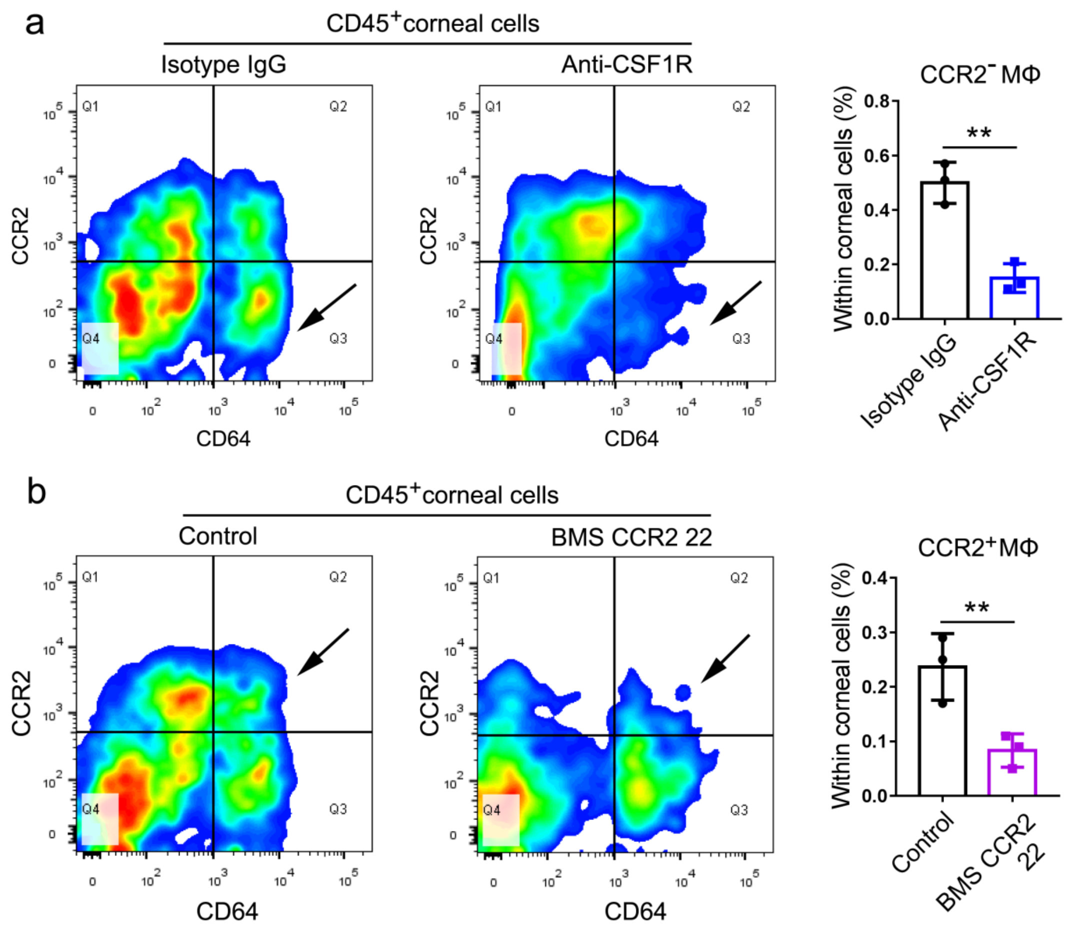

Supplementary Figure 7. Depletion of corneal macrophages. (**a**) Flow cytometric analysis of

corneal cells from isotype IgG and anti-CSF1R antibody-treated mice at P28 (*n* = 3 independent experiments, 10 mice per experiment in each group). (**b**) Flow cytometric analysis of corneal cells from control and BMS CCR2 22-treated mice at P28 (*n* = 3 independent experiments, 10 mice per experiment in each group). **P < 0.01

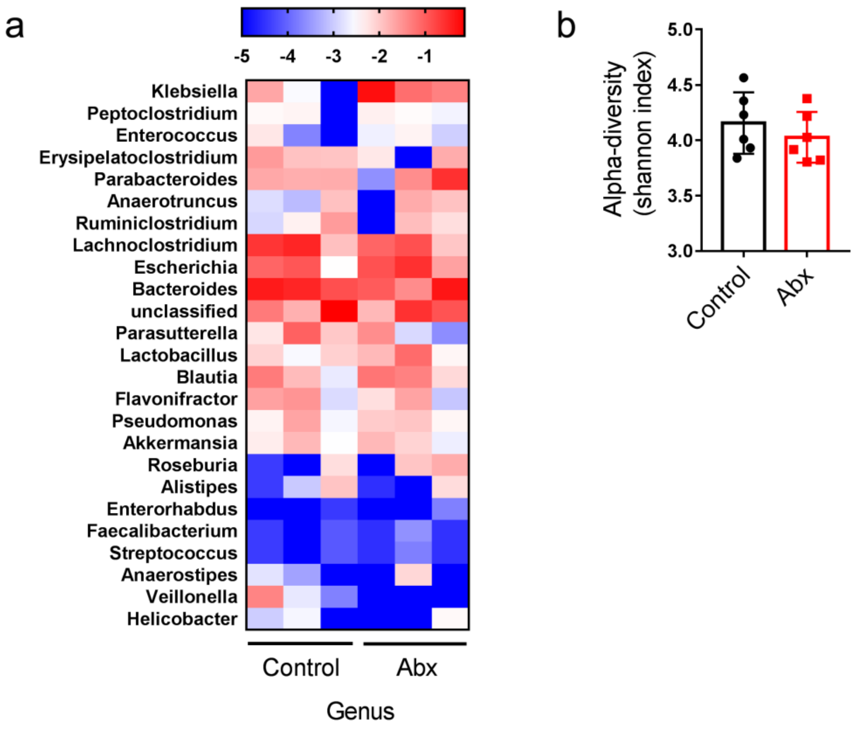

Supplementary Figure 8. Investigation of the diversity and abundance of gut microbiota in control and antibiotic-treated mice at postnatal 8 weeks with 16S rRNA sequencing. (**a**) Heat map of gut microbiota at the genus level in the two groups of mice (*n* = 3 mice in each group). (**b**) Shannon indices of gut microbiota in the two groups of mice (*n* = 3 mice in each group).

Gene	A_P28_1	A_P28_2	A_P28_3	C_P28_1	C_P28_2	C_P28_3
Gm29609	40.807	57.03897	46.2797	456.0914	417.2933	530.1556
Aadac	61.6269	26.40693	31.76058	482.5498	285.8048	235.0594
Aak1	448.0442	831.2902	622.5073	7496.53	6249.355	554.5774
Sptlc3	6.662367	7.393941	7.259561	70.55558	41.09015	30.52719
Cxcr2	2.498388	2.112554	9.074451	15.11905	16.43606	35.61506
Abi2	203.2022	183.7922	206.8975	1984.376	105.9213	617.6669
Tead1	373.0926	474.2685	385.6642	1606.399	1932.15	1922.196
Ggnbp1	59.12851	186.9611	162.4327	633.7403	471.1671	662.4401
Kbtbd8	113.2602	58.09525	49.00204	417.0339	277.5868	262.5339
Adam23	42.47259	28.51949	17.24146	156.2302	37.43769	181.128
Vnn3	1.665592	8.450218	1.81489	17.63889	18.26229	12.21088
Mmp10	4.163979	13.7316	5.444671	41.57739	21.00163	31.54477
Kynu	43.30539	24.29438	19.96379	158.7501	88.5721	109.8979
Fyttd1	5753.787	2254.096	1695.107	19653.51	9424.254	9610.978
Smarca1	306.4689	211.2554	257.7144	1510.645	762.4506	775.3907
Ptgdr	4.163979	6.337663	14.51912	37.79763	29.21966	29.50962
Ndnf	204.8678	164.7792	153.3582	970.1392	528.6933	500.646
Nr4a3	36.64302	15.84416	36.2978	146.1508	98.61636	96.66945
Adam21	4.996775	5.281386	1.81489	16.37897	10.04426	19.33389
Ptp4a1	988.5287	799.6019	875.6845	4146.4	2208.824	3697.861
Skp1a	4928.486	6854.183	3794.935	27637.63	14970.51	14639.82
Nfat5	1885.45	1905.524	1856.633	4369.406	2951.186	13319.01
Taf13	1097.625	874.5975	813.9783	6240.389	1945.847	1982.232
Sult1b1	40.807	13.7316	30.85313	178.9088	66.65736	67.15983
Ar	161.5624	106.684	137.9317	723.1947	336.9392	408.0468
Sptssb	168.2248	78.16451	142.4689	603.5022	423.6851	360.2209
Cubn	61.6269	89.78356	86.20729	352.7779	225.5393	266.6042
Akr1c18	66.62367	49.64503	58.07649	254.5041	199.059	166.882
Fut9	134.0801	108.7966	105.2636	468.6906	399.9441	355.133
Pus10	817.8056	803.827	735.938	4274.912	1482.898	2449.299

Supplemental table 1. FPKM value of genes used in the heat map of metabolic process. A_P28: Abx_treated mice at P28; C_P28: Control mice at P28.

Gene	A_P28_1	A_P28_2	A_P28_3	C_P28_1	C_P28_2	C_P28_3
Anapc10	278.9866	229.2122	182.3965	1122.59	630.9621	595.2803
Samd9l	6750.643	4702.546	5366.63	27389.42	14996.08	13505.23
Ccne2	460.5361	242.9438	227.7687	1315.358	804.4539	687.8794
Hepacam2	4.996775	10.56277	8.167006	35.27779	15.52295	18.31632
Sycp2	20.8199	6.337663	15.42657	46.61708	38.35081	38.66778
Ppp1cb	10383.3	9611.067	9614.381	40168.8	21049.11	23788.82
Haus3	382.2533	287.3074	313.976	1341.816	720.4473	733.6702
Itgb3bp	254.0027	189.0736	189.656	836.5876	505.8654	451.8025
Ccng1	4276.407	3576.555	3649.744	15296.7	8538.533	8489.613
Cetn2	1307.49	1054.165	1038.117	4525.636	2585.027	2334.313
Top1	4130.668	3185.732	2960.086	12289.27	7615.375	8343.082
Phip	6432.515	4734.235	5087.137	20571.99	12971.7	10978.6
Hells	1059.316	511.2382	539.0224	2516.062	1534.945	1659.662
Usp9x	6713.168	5735.585	5846.669	23455.95	12296	13293.58
Mis12	908.5803	862.9785	662.4349	2906.638	1520.336	2042.269
Rbbp8	1230.872	939.0305	825.7751	3699.128	2200.606	2050.41
Gnai3	9824.493	7603.083	7563.555	31332.98	16777.57	17941.85
Dynlt3	7010.476	5677.49	5299.479	22038.54	13067.58	12401.16
Wapl	4803.567	4003.291	3642.485	15001.88	8147.72	9352.515
Arl8b	4132.333	3471.983	3287.674	12886.47	7066.593	8239.29
Cetn3	1706.399	1349.922	1339.389	5319.387	3089.979	2922.47
Klhl9	4077.369	3128.693	3449.199	12148.16	7409.924	7571.762
Zfp449	263.9963	205.9741	171.5071	786.1907	434.6425	409.0644
Actr3	9936.088	8437.543	7998.221	26693.95	22300.99	17922.52
Lrrcc1	1289.168	929.524	695.103	3495.021	2129.383	1760.402
Gnl3	966.876	671.7923	669.6945	2450.546	1657.303	1741.068
Vps4b	5638.028	4641.282	4242.306	15946.82	9712.799	10975.54
Nek1	1055.985	891.498	913.7972	3253.116	1934.89	2003.601
Chmp2b	5378.196	4539.88	3955.553	16137.07	8734.853	9809.405
Stag2	7843.272	6383.083	6381.154	24266.08	13623.67	13227.43

Supplemental table 2. FPKM value of genes used in the heat map of cell division. A_P28: Abx_treated mice at P28; C_P28: Control mice at P28.

Gene	A_P28_1	A_P28_2	A_P28_3	C_P28_1	C_P28_2	C_P28_3
Ndnf	204.8678	164.7792	153.3582	970.1392	528.6933	500.646
Cav1	965.2104	613.6971	515.4288	1092.352	423.6851	5587.494
Ccr2	129.9162	102.4589	177.8592	563.1847	281.2393	391.7657
Fap	797.8185	558.7707	708.7146	3067.908	1429.937	1667.802
Hif1a	9245.7	7240.78	6863.007	30496.39	15341.24	20151
Il18	1129.271	852.4157	817.608	3782.283	2064.552	2029.041
Nat1	16.65592	9.506495	9.074451	55.43653	21.91475	19.33389
Pdcd10	2832.339	2373.455	2225.055	9817.305	5360.895	4882.316
Pten	5611.379	4792.33	4302.197	17761.11	9251.676	12034.84
Nus1	1688.077	1612.935	1433.763	5405.061	3125.591	3922.744
Rspo3	11.65914	15.84416	21.77868	42.83732	51.13441	33.57991
C1galt1	867.7733	757.3508	847.5537	3027.59	1645.432	1646.433
Ereg	1658.929	1467.169	1315.795	4927.551	3295.43	2988.612
Naa15	7336.099	5427.152	5179.697	20709.32	12132.55	12428.64
Pde3b	157.3984	107.7403	134.3019	467.4307	271.195	264.569
Scg2	48.30216	43.30737	18.1489	97.01392	94.05079	80.38828
Ppp3r1	4123.172	3621.975	3382.955	12369.9	6735.132	7747.802
Cdc42	22455.51	17936.64	17378.48	61761.33	35817.83	38365.56
Epgn	1514.856	1135.498	816.7006	3671.41	2188.735	2052.445
Casp8	1603.965	1195.706	1186.031	3804.962	2567.678	2581.583
Sirt1	911.0787	768.9698	676.9541	2474.485	1316.711	1502.956
Ccl12	31.64624	16.90044	24.50102	71.8155	36.52458	54.94895
Foxc1	1267.515	1733.351	1274.053	3758.344	2211.563	3577.787
Pik3r3	2543.359	1934.044	1946.47	5372.303	4394.82	4291.106
Rtn4	1926.257	1705.888	1433.763	4244.674	2682.73	4048.923
Krit1	2041.183	1531.602	1416.522	4450.041	3185.856	3110.721
Calcrl	1517.354	1054.165	1118.88	3618.493	2268.176	2006.654
Angpt1	724.5324	716.156	628.8595	2209.902	1172.439	1036.907
Prkx	1933.752	1599.204	1588.936	4321.529	2902.791	3645.965
Adtrp	2450.085	1997.42	1913.802	5532.313	3906.304	4050.959

Supplemental table 3. FPKM value of genes used in the heat map of angiogenesis. A_P28: Abx_treated mice at P28; C_P28: Control mice at P28.

Gene	A_P28_1	A_P28_2	A_P28_3	C_P28_1	C_P28_2	C_P28_3
S100a8	3.331184	7.393941	4.537226	148.6707	24.65409	105.8276
Rnf17	32.47904	19.01299	18.1489	219.2263	60.26556	74.28284
Igf1	1377.444	1025.645	1371.15	6377.72	3283.56	2901.101
Glyat	92.44034	82.38962	49.00204	309.9406	205.4508	185.1983
Bcl2a1b	20.8199	19.01299	18.1489	66.77582	55.69998	55.96652
S100a9	310.6329	153.1602	196.0081	685.3971	538.7375	765.215
Echdc1	1080.969	874.5975	925.594	4151.44	2109.294	2068.726
Hif1a	9245.7	7240.78	6863.007	30496.39	15341.24	20151
Uqcrb	1867.961	1288.658	1419.244	6601.986	3056.194	3198.232
mt-Atp6	61735.99	45851.94	49428.54	223363.8	111849.2	103211.4
mt-Atp8	14528.96	11378.22	12361.22	53082.99	26736.9	25163.57
Copb1	6829.759	5388.07	5135.232	22325.8	12039.41	12899.77
mt-Nd3	3733.424	2495.983	2952.826	13549.19	5633.003	5246.607
Yme1l1	5784.6	4436.364	4155.191	18088.69	10191.27	9806.352
Tmlhe	525.4942	372.8659	306.7164	1407.332	882.9817	875.1129
Ube2d2a	2888.136	2524.503	2220.518	9459.487	4755.5	5729.954
Abcb7	2313.507	1810.459	1963.711	7730.876	4197.587	3867.795
Spaca1	149.0705	109.8528	117.9679	488.8494	224.6262	253.3757
Casp8ap2	1117.612	708.762	771.3283	2983.493	1892.886	1686.119
Crls1	566.3012	494.3377	405.628	1538.364	1028.167	1128.489
Slc25a32	517.999	406.6667	409.2577	1369.534	853.762	1125.436
Dnaja1	2970.583	2742.096	2490.029	9269.239	5808.321	5419.594
Cox5a	839.4582	605.2469	685.1211	2740.328	1240.923	1316.74
Bnip3	398.0764	398.2165	421.962	1404.812	830.0211	773.3556
Rars2	1202.557	932.6928	961.8918	3054.049	2343.052	2217.292
Hibch	706.2109	587.2901	566.2458	2019.653	1156.003	1375.759
Dnm1l	5179.99	4045.542	4074.429	14138.83	9077.271	9296.548
Fzd3	65.79087	40.13853	39.02014	162.5298	66.65736	126.1791
Ppp3r1	4123.172	3621.975	3382.955	12369.9	6735.132	7747.802
Hspd1	3831.694	3031.516	3269.525	9798.406	6553.423	7964.545

Supplemental table 4. FPKM value of genes used in the heat map of mitochondria. A_P28: Abx_treated mice at P28; C_P28: Control mice at P28.

Gene	A_P28_1	A_P28_2	A_P28_3	C_P28_1	C_P28_2	C_P28_3
Nrg1	522.9958	510.1819	356.6259	1349.375	1050.082	1204.807
Grin3a	135.7457	63.37663	98.00407	361.5973	284.8917	424.328
P2ry1	462.2017	366.5282	387.4791	1275.04	893.9391	892.4116
Tlr4	1110.117	857.6971	922.8717	3719.287	2401.491	2055.498
Arf4	6491.644	4895.845	5332.147	20786.18	12249.43	12942.51
Igf1	1377.444	1025.645	1371.15	6377.72	3283.56	2901.101
Kras	2682.436	2354.442	2067.16	6271.887	4584.748	4723.574
Hif1a	9245.7	7240.78	6863.007	30496.39	15341.24	20151
Pafah1b1	8537.823	6793.975	6671.536	21204.47	13927.74	15417.25
Ccdc66	658.7415	483.775	515.4288	1382.133	1092.998	1120.348
Pten	5611.379	4792.33	4302.197	17761.11	9251.676	12034.84
Hexb	5648.022	5008.867	4935.594	13699.12	9443.43	10087.2
Dlg2	1248.361	1334.078	923.7791	4133.801	2426.145	2588.706
Nr4a3	36.64302	15.84416	36.2978	146.1508	98.61636	96.66945
Uba6	3571.029	2610.061	2588.033	11412.36	7071.158	5794.061
Gabrb2	46.63657	30.63204	37.20525	177.6489	113.2262	92.59915
Ccr2	129.9162	102.4589	177.8592	563.1847	281.2393	391.7657
Alms1	415.5651	266.1819	392.0163	973.919	700.3588	782.5137
Nbn	927.7346	682.3551	566.2458	2053.671	1422.632	1569.098
Chl1	6084.407	3602.962	3390.215	11790.34	14007.18	8455.015
Slitrk6	821.1367	576.7274	636.119	1999.495	1369.672	1263.826
Nipbl	4355.522	3921.957	3740.489	10133.55	6878.491	7106.731
Cnr1	78.28281	57.03897	67.15094	190.2481	137.8803	146.5305
Syt4	639.5872	521.801	784.0326	2053.671	1188.875	1323.863
Braf	4467.95	3940.97	3415.623	10066.77	6541.552	7336.702
Mapk1	4851.869	4159.62	5643.401	11029.35	7578.85	10747.61
Sema5a	3964.108	3526.91	3206.911	9967.235	5686.877	7087.397
Picalm	7068.771	5633.126	4147.932	16896.8	9890.856	11434.47
Slc4a7	1542.338	1048.883	1100.731	3293.434	2037.158	2241.714
Bche	158.2312	76.05196	134.3019	471.2105	235.5835	245.2351

Supplemental table 5. FPKM value of genes used in the heat map of neurogenesis. A_P28: Abx_treated mice at P28; C_P28: Control mice at P28.

Gene	A_P28_1	A_P28_2	A_P28_3	C_P28_1	C_P28_2	C_P28_3
Clec4e	4.163979	0	3.62978	8.819447	8.21803	21.36904
Cd300lf	6.662367	2.112554	1.81489	21.41866	12.7836	11.1933
Clec7a	52.46614	31.68832	49.00204	224.2659	151.577	124.1439
Ccr5	72.45324	62.32036	78.04028	331.3592	178.0573	223.8661
Gbp4	399.742	250.3377	244.1027	1344.336	1050.995	602.4033
Mmp12	50.80055	42.25109	53.53926	225.5259	119.618	144.4954
Clec5a	31.64624	28.51949	26.31591	131.0318	55.69998	96.66945
Ifi204	1322.48	896.7794	922.8717	4554.615	2732.952	2754.57
Nr1d2	2729.905	2023.827	2416.526	10105.83	6514.159	5962.979
Cd180	34.14463	17.95671	43.55737	118.4326	85.83276	95.65187
Ccr2	129.9162	102.4589	177.8592	563.1847	281.2393	391.7657
Vav3	973.5384	868.2599	763.1613	3355.17	2170.473	2275.294
Il1b	7.495163	15.84416	18.1489	34.01787	43.82949	44.77322
Tlr4	1110.117	857.6971	922.8717	3719.287	2401.491	2055.498
Il18	1129.271	852.4157	817.608	3782.283	2064.552	2029.041
Rasgrp1	625.4297	564.052	449.1853	1605.139	1198.919	1494.815
Prdx1	10519.04	6077.819	7627.984	26698.99	18566.36	17720.02
Tpd52	6114.387	6914.391	3280.414	18964.33	10725.44	11845.57
Sp100	448.877	293.6451	352.9961	1258.661	688.4883	844.5857
Cd36	121.5882	115.1342	154.2657	454.8315	297.6753	236.077
Cd84	57.46292	32.74459	77.13283	175.129	128.7491	117.0209
Ptpn2	914.4099	690.8053	558.0787	2228.8	1534.032	1543.658
Mnda	122.421	82.38962	105.2636	343.9584	210.0163	199.4443
Evi2b	62.45969	34.85715	44.46481	161.2699	120.5311	60.03681
Hspd1	3831.694	3031.516	3269.525	9798.406	6553.423	7964.545
Irgm1	1240.866	927.4114	1130.677	3447.144	2205.171	2259.012
Hmgb1	6286.776	5038.442	4500.928	17520.46	10126.44	10155.38
Msr1	78.28281	57.03897	81.67006	243.1648	121.4442	150.6008
Lair1	54.96453	68.65802	34.48291	185.2084	93.13768	93.61673
Rb1	1673.92	1478.788	1361.168	4577.293	2773.129	3206.373
Casp8	1603.965	1195.706	1186.031	3804.962	2567.678	2581.583
Sirt1	911.0787	768.9698	676.9541	2474.485	1316.711	1502.956
Mcl1	7110.411	7034.806	6067.178	18459.1	11772.78	14671.37
Ccl12	31.64624	16.90044	24.50102	71.8155	36.52458	54.94895
Tra2b	3474.424	2806.529	2693.297	8989.537	5155.444	5682.128
Stap1	41.63979	39.08226	24.50102	122.2123	48.39507	60.03681
Rtn4	1926.257	1705.888	1433.763	4244.674	2682.73	4048.923
Tnfsf13	215.6941	61.26408	85.29984	317.5001	277.5868	191.3037
Steap4	3710.938	3084.33	2860.267	6773.336	6159.87	7964.545
Cd44	13196.48	10715.93	9096.23	30375.44	19793.58	20952.85
Vcam1	1678.084	1451.325	1417.429	3515.18	2726.56	3533.014
Vcam1	1678.084	1451.325	1417.429	3515.18	2726.56	3533.014
Serpinb2	46.63657	23.2381	29.94569	85.67463	71.22293	59.01924
C3ar1	177.3855	161.6104	225.9538	511.5279	304.0671	394.8184
Angpt1	724.5324	716.156	628.8595	2209.902	1172.439	1036.907
Il4	17.48871	20.06927	15.42657	41.57739	37.43769	33.57991
Plcb1	165.7264	164.7792	133.3944	384.2759	258.4114	338.8519
Jak2	2878.975	2586.823	2153.367	6273.147	4411.256	5047.163
Ddx20	762.0082	614.7533	590.7468	1631.598	1206.224	1221.088
Rc3h1	2141.951	1926.65	1817.613	5199.694	3209.597	3679.544
Epsti1	78.28281	61.26408	59.89138	139.8512	109.5737	159.759
Ywhaz	21641.03	14328.4	12455.59	43983.84	24488.82	29706.01
Havcr2	45.80377	48.58875	39.02014	95.754	67.57047	104.81

Mapk1	4851.869	4159.62	5643.401	11029.35	7578.85	10747.61

Supplemental table 6. FPKM value of genes used in the heat map of macrophage. A_P28: Abx_treated mice at P28; C_P28: Control mice at P28.
